# Purkinje Cells in Crus I and II Encode the Visual Stimulus and the Impending Choice as Monkeys Learn a Reinforcement-Based Visuomotor Association Task

**DOI:** 10.1523/JNEUROSCI.0082-25.2026

**Published:** 2026-08-31

**Authors:** Anna E. Ipata, Valeria Fascianelli, Chris I. De Zeeuw, Naveen Sendhilnathan, Stefano Fusi, Michael E. Goldberg

**Affiliations:** ^1^Department of Neuroscience, Columbia University, New York, New York 10027; ^2^Mahoney Center for Brain and Behavior Research, Columbia University, New York, New York 10027; ^3^Center for Theoretical Neuroscience, Columbia University, New York, New York 10027; ^4^Department of Neuroscience, Erasmus MC, Rotterdam 3015, The Netherlands; ^5^Netherlands Institute for Neuroscience, Royal Dutch Academy of Arts and Science (KNAW), Amsterdam 1105, The Netherlands; ^6^Kavli Institute for Brain Science, Columbia University, New York, New York 10027; ^7^Zuckerman Mind, Brain, and Behavior Institute, Columbia University, New York, New York 10027; ^8^Department of Neurology, Psychiatry, and Ophthalmology, Columbia University College of Physicians and Surgeons, New York, New York 10027

**Keywords:** cerebellum, Purkinje cells, reinforcement learning, visuomotor association task

## Abstract

Evidence implicates the cerebellum in motor learning and performance and relatively simple sensorimotor associations such as classical conditioning. However, little is known about how Purkinje cells encode more complex associations, such as arbitrary stimulus–response mappings. To address this, we recorded from Crus I/II Purkinje cells while two male macaques learned to associate novel fractal cues with left- or right-hand bar release. Here we show that as learning progresses, the simple spike activity of individual neurons becomes more selective for stimulus–response associations, with selectivity for left or right association developing closer to the appearance of visual stimuli. Initially, most neurons respond to both associations, irrespective of the identity of the stimulus and the associated movement, but as learning advances, more neurons distinguish between specific stimulus-hand associations. Moreover, complex spikes, which respond after the appearance of the stimuli, decrease their activity as the monkeys learn the task, suggesting that climbing-fiber activity encodes higher-order sensorimotor associations beyond mere error signaling. Using a linear decoder, we found that in early learning stages, the visual stimulus can be decoded only when the choice can also be decoded. As learning progresses, the visual stimulus is decoded earlier than the choice. A simple model can replicate the observed Purkinje cell signals in both the early and late learning stages.

## Significance Statement

The cerebellum is traditionally associated with motor learning, yet its contribution to complex stimulus–response associations remains unclear. Recording Purkinje cells in Crus I/II as monkeys learned visuomotor associations, we found that activity evolves from reactive to predictive with learning. Simple spikes encode the upcoming choice progressively earlier, while complex spikes shift from a reactive response to the stimulus during early learning to a predictive signal before movement. Thus, cerebellar populations integrate sensory and motor inputs to generate anticipatory, decision-related activity.

## Introduction

The ability to transform sensory inputs into directed actions requires integrating sensory information with contextual cues to generate appropriate motor responses. Stimulus–response association tasks have become a cornerstone of neuroscience for understanding how sensory signals are translated into actions. Goal-directed behavior depends on the coordinated activity of cortical regions, including premotor (Buch et al., 2006; Hoshi and Tanji, 2006; Wang et al., 2019), parietal cortex (Shadlen and Newsome, 2001), and prefrontal cortex (Tanji and Hoshi, 2001; Pasupathy and Miller, 2005; Histed et al., 2009; Yamagata et al., 2012; Nakayama et al., 2015) together with subcortical structures (Pasupathy and Miller, 2005; Histed et al., 2009; Arimura et al., 2013; Hoshi, 2013).

The cerebellum has long been recognized as a “machine for learning,” but whether it also contributes to transforming sensory information into context-appropriate actions remains debated. Anatomical tracing studies show that the posterolateral cerebellum is reciprocally connected with the same cortical and subcortical regions involved in associative learning, such as prefrontal, premotor, and sensorimotor cortices via deep cerebellar nuclei and thalamic relays (Middleton and Strick, 2001; Kelly and Strick, 2003; Akkal et al., 2007; Prevosto et al., 2010; Bostan et al., 2013; Pisano et al., 2021; Sendhilnathan et al., 2024) as well as with subcortical areas. These bidirectional pathways suggest that the lateral cerebellum participates in higher-order associative processing (Caligiore et al., 2017).

Classical sensory motor association paradigms such as delayed eyeblink conditioning (Ohmae and Medina, 2015; ten Brinke et al., 2015; Silva et al., 2024), vestibulo-ocular reflex adaptation (Wulff et al., 2009; Dash et al., 2013), smooth pursuit adaptation (Medina and Lisberger, 2009; Li et al., 2011; Dash et al., 2013), whisker reflex adaptation (Romano et al., 2018), and saccadic adaptation (Medina and Lisberger, 2008; Herzfeld et al., 2018) have shown that Purkinje cell (PCs) simple (SSs) and complex spikes (CSs) activity adjusts trial-by-trial to optimize the timing and kinematics of movements.

More recent electrophysiological and inactivation studies in mice and monkeys (Deverett et al., 2018; Chabrol et al., 2019; Deverett et al., 2019; Wagner et al., 2019; Sendhilnathan et al., 2020; Bina et al., 2021; Verpeut et al., 2023; Brown et al., 2024; Sendhilnathan et al., 2024; Flierman et al., 2025) demonstrate that SSs encode not only conditioned motor responses but also arbitrary cue–response mappings and decision variables. However, whether cerebellar cortical activity remains essential once a task is well learned remains debated: while some studies report that overtrained performance is spared after inactivation (Timmann et al., 2002; Sendhilnathan et al., 2024), others find persistent deficits (Burguière et al., 2010; Deverett et al., 2019).

The modulation of SS activity is shaped by CSs (Badura et al., 2013), which induce plasticity at parallel fiber→PC synapses (Lisberger, 2021). CSs convey diverse signals, including temporal-difference prediction error (Ohmae and Medina, 2015; Heffley et al., 2018), reward expectation (Kostadinov et al., 2019), instructive teaching signals (Silva et al., 2024), learning-dependent predictive information (Bina et al., 2021), trial onset prediction (Sendhilnathan et al., 2021), and prediction of upcoming position errors and kinematic (De Gruijl et al., 2014; Streng et al., 2017; Gaffield et al., 2022; Muller et al., 2023).

Despite growing evidence for cerebellar involvement in associative learning, it remains unclear whether Purkinje cells are selective for one association and if this selectivity depends on learning. Determining whether such selectivity emerges gradually and shifts in time during task acquisition is essential for understanding how cerebellar circuits contribute to flexible behavior and implement forward internal models linking sensory cues to predicted actions.

To address these issues, we reanalyzed SS and CS activity from PCs recorded previously in Crus I/II of nonhuman primates performing a visuomotor association task (Sendhilnathan et al., 2020). As monkeys learned stimulus–movement associations, PC activity shifted from reactive to predictive: more PCs encoded hand choice, selectivity emerged earlier in the trial, and population decoding confirmed that both stimulus identity and upcoming response were represented progressively sooner with learning. This supports a role for PCs in integrating sensory and motor information to generate predictive signals, extending the cerebellum’s forward model function beyond motor control.

## Materials and Methods

### Behavioral tasks

The monkeys were seated in a Crist primate chair, positioned inside a dimly lit recording booth with their heads securely immobilized. The visual stimuli, generated by a Hitachi CP-X275 LCD projector, were presented to the monkeys on a back-projection screen positioned in front of the monkeys, at a distance of 57 cm. A Dell PC running the NIH VEX graphic system controlled the Hitachi CP-X275 LCD projector. We used the NIH REX system for behavioral control (Hays et al., 1982). Each trial began with the monkey grasping two bars with the left and the right hands (Fig. 1*A*). Following this, the trial initiation cue (a 1° × 1° white square) was presented for 800 ms. Subsequently, one of the two stimuli appeared either briefly for 200 ms or remained visible until the monkey initiated a hand response by releasing one of the bars. The stimulus presentation indicated which hand the monkey should release: one stimulus for the left hand and another for the right. A correct response, releasing the hand associated with the presented stimulus, was immediately rewarded with a drop of juice, with a delay of 1 s following the initiation of the correct hand movement. The monkeys have up to 3 s to respond after the stimulus appears. From the initiation of the hand movement, a 700 ms delay (intertrial interval, ITI) was enforced before the commencement of the next trial. Trials where no hands or both hands were released were aborted automatically.

For each neuron, recordings began while the monkeys performed a well-learned visuomotor association task (overtrained, OT), in which performance was consistently above chance (90–100%). After ∼30 trials, two novel visual stimuli (fractals) that the monkeys had never encountered before were introduced, initiating a new learning condition (new task, NT). After performance reached ∼80% correct, the stimulus–response associations were reversed (REV), requiring the monkey to learn the new associations. A session corresponded to a continuous recording from a single, well-isolated Purkinje cell, typically lasting ∼15 min.

### Data analysis of behavioral performance

We quantified the progression from chance level to proficient performance using binomial logistic regression on trial-by-trial accuracy. For each session and condition, we modeled the probability that a response on trial *t* was correct (1) versus incorrect (0) as a function of the trial’s ordinal index. For each representative session, behavioral performance was fit using a binomial generalized linear model in MATLAB:

$$\mathrm{logit}(\;p_{i})=\log\left[\frac{\;p_{i}}{\left(1-p_{i}\right)}\right]=\beta_{0}+\beta_{1}x_{i},$$

where *p*_i_ is the predicted probability of a correct response on trial *i*, *x_i_* is the ordinal trial number, *β*_0_ is the intercept, and *β*_1_ is the slope. The two parameters, *β*_0_ and *β*_1_, were estimated by maximum likelihood using MATLAB (fitglm in MATLAB, Distribution=binomial).

Model fit was evaluated by comparing the fitted model to a constant-accuracy (null) model via a likelihood-ratio *χ*^2^ test, and the fitted model was used to obtain predicted probabilities for each trial. Unlike fixed-width binning, which averages over blocks of trials and can blur abrupt changes in performance, the logistic-regression approach provides a continuous, trial-resolved estimate of accuracy. This allows the precise identification of the single trial at which performance shifts from the learning phase to the learned phase, even when the change occurs rapidly within a small number of trials.

The transition trial in NT was defined as the first trial with a predicted probability ≥0.80. Trials with probability <0.80 were labeled early learning (EL); those with probability ≥0.80 were labeled late learning (LL). If no trial reached 0.80, all trials were considered EL; if all trials exceeded 0.80 from the start, the session was considered LL throughout. The 0.80 criterion was chosen a priori to reflect near-asymptotic performance.

### Recording Purkinje cell activity

We recorded from the right side of Crus I and Crus II in two male rhesus macaques (*Macaca mulatta*). For daily recordings, we utilized glass-coated tungsten electrodes with an impedance range of 0.8–1.2 MOhms (FHC). These electrodes were introduced into the target cerebellar region using a Hitachi microdrive system. The raw signal captured by the electrode was initially processed through a Neurocraft head stage and an amplifier provided by FHC. To isolate the frequencies of interest, the signal underwent bandpass filtering using a Krohn-Hite filter, with a low-pass setting at 300 Hz and a high-pass setting at 10 kHz, employing a Butterworth filter configuration. The filtered signal was then digitized and processed through a Micro1401 system (Cambridge Electronic Design). The neural data acquisition was integrated with the NEI REX-VEX system, coupled with the Spike2 platform, to ensure synchronized collection of event-related and neural activity data. This setup allowed for real-time monitoring of neural signals and task events, crucial for aligning neuronal responses with specific behavioral outcomes. In both online (during recordings) and offline (postexperiment) analyses, we focused on identifying cerebellar PCs, characterized by their distinct complex and simple spike activity patterns.

Only neurons with stable isolation throughout the recording session were included in the analysis. Stability was assessed during offline spike sorting by continuously inspecting the SS and CS waveforms across the entire session. For each unit, spikes were accepted only when they formed a clearly isolated cluster, distinct from background noise and from other units, and when the waveform shape and amplitude remained consistent over time. In practice, we compared SS and CS waveforms from the beginning, middle, and end of the recording session and verified that there were no abrupt changes in waveform shape, peak-to-peak amplitude, or baseline noise level that would indicate electrode drift or loss of isolation. The signal-to-noise ratio was also inspected throughout the session to ensure reliable discrimination of spikes from noise. Units in which the waveform became unstable, the spike cluster merged with noise or another unit, or SS/CS identification became ambiguous were excluded from further analysis. For example, when the climbing-fiber pause started to fill up during the recording session with SSs from another unit, the PC involved was discarded.

### Quantification and statistical analyses of simple spikes

#### Baseline-normalized *z*-score time course

For each neuron we computed the firing rate by convolving spikes with a Gaussian kernel (*σ* = 15 ms), in two time windows: from −100 to 400 ms from the onset of the stimulus and from 400 ms before to 400 ms after the onset of the movement, and then we averaged its spike-density firing rate across trials. The baseline was calculated as the mean and standard deviation of the firing rate over the 100 ms preceding stimulus onset. We then divided the average firing rate into overlapping 80 ms bins with 10 ms increments and computed the mean firing rate in each segment. For each bin, we converted the mean rate into an absolute *z*-score deviation from the baseline to capture both increases and decreases in firing relative to the baseline.

#### SSs modulation from baseline to stimulus–response interval

For each neuron, and for each condition (OT, EL, and LL), we estimated the earliest time at which activity departed from baseline separately for left- and right-hand trials*.* Because during the early learning subjects often perseverated with one hand—resulting in highly imbalanced left- and right-response trials—we required a minimum of five correct trials in each response condition before including a neuron in our analysis. Spike-density functions (SDFs) were computed per trial by convolving spikes with a Gaussian kernel (*σ* = 15 ms). The baseline distribution was defined as the mean SDF in the 100 ms immediately before stimulus onset. We then scanned the postevent epoch with sliding 80 ms windows advanced in 10 ms steps. In every window, we computed each trial's mean SDF and compared the window's distribution to baseline with a two-sample Kolmogorov–Smirnov test (MATLAB kstest2). Because each neuron is tested in many overlapping windows, multiple comparisons inflate false-positive risk; therefore, Benjamini–Hochberg false discovery rate (FDR) correction (MATLAB mafdr, BHFDR=true) was applied across all windows for that neuron, hand, and epoch. A window was considered significant if pFDR < 0.05. To guard against isolated positives, significance required ≥6 consecutive FDR-significant windows, and the latency was defined as the center time of the first window in that run. As a robustness check on the choice of test, we repeated the sliding-window procedure using Welch’s unequal-variance *t* test (MATLAB ttest2, Vartype=“unequal”) and the Brunner–Munzel test (nonparametric, robust to unequal variances; implemented in MATLAB). For both alternatives, we again applied BH-FDR across windows and used the same ≥6-bin criterion; results were consistent with the KS-based analysis.

#### SSs neuronal population: from baseline to stimulus–response interval

After computing each neuron’s response-onset latency, we used a sliding-threshold *χ*^2^ sweep to determine when the proportion of neurons showing a left or right significant response diverges across three learning stages (OT, EL, LL). We defined threshold times every 80 ms across two windows: 0–300 ms after stimulus onset and −300 to +400 ms around movement onset. At each threshold, we counted, for each learning stage, how many neurons had their individual response time at or before that threshold versus those that did not. For each pairwise comparison of two stages, these counts formed a 2 × 2 contingency table which we tested using Pearson's chi-square test to obtain a raw *p* value. Repeating this across all thresholds yielded a time series of raw *p* values for each of the three stage pairs. To account for the multiple, overlapping threshold tests, we applied the Benjamini–Hochberg procedure separately to each series of raw *p* values. We then recorded for each comparison, the earliest threshold time at which the raw *χ*^2^ *p* value fell below 0.05 in at least two consecutive significant bins and the earliest threshold time at which the FDR-adjusted *p* value fell below 0.05 in at least two consecutive bins. To ensure that any identified divergence was not an artifact of multiple testing or uneven responder counts, we performed a permutation test. We pooled all neuron response times (including nonresponders), randomly reshuffled the condition labels, and reassigned entries back into the three stage groups in proportion to their original sizes. For each permutation, we repeated the entire sliding-threshold *χ*^2^ sweep, capturing both the minimum *p* value achieved across all thresholds and the first threshold time at which *p* dropped below 0.05. By comparing our observed minimum *p* value and first-significance time to the distribution from these permutations, we derived empirical *p* values. A result was deemed permutation significant only if fewer than 5% of permutations produced an equal or more extreme outcome.

#### SSs single-neuron SR-left versus SR-right selectivity

We defined selectivity for a neuron as the presence of a significant difference in firing rate between stimulus–response (SR) left and SR-right trials at any time during the trial.

Spike trains were converted to trial-wise SDFs (Gaussian kernel, *σ* = 15 ms). We included only conditions with at least five correct trials in both SR-left and SR-right associations. Analyses were run separately in stimulus-locked and movement-locked frames using sliding 80 ms windows advanced in 10 ms steps. Within each window we averaged the SDF across time on every trial to obtain two distributions of windowed activity. In every window we compared the two sets of trials with a two-sample Kolmogorov–Smirnov test (MATLAB kstest2), yielding a raw *p* value. When the number of trials differed between SR-left and SR-right trials, we applied a balanced resampling to remove sample-size leverage. Group sizes were equalized by sampling *N* trials per condition, where *N* was the smaller SR-left and SR-right number of trials. We randomly drew *N* trials without replacement from each condition and computed the KS statistic. We then formed an empirical null by shuffling SR-left/SR-right labels 1,000 times (MATLAB randperm/randsample); on each shuffle, we again drew *N* trials per group and recomputed the KS distance. The permutation *p* value was the fraction of shuffled distances ≥ the observed (with *a* + 1 correction). Because many overlapping windows were tested, we applied Benjamini–Hochberg FDR correction (MATLAB mafdr, BHFDR=true, *q* < 0.05) across all windows and performed separately for each *p*-series (the KS series and the balanced-permutation series). To guard against isolated positives, significance required ≥6 consecutive FDR-significant windows; the discrimination latency was defined as the center time of the first window in that run. Finally, to ensure conclusions did not hinge on the choice of test, we repeated the sliding-window analysis with Welch’s unequal-variance *t* test (ttest2, Vartype=“unequal”) and the Brunner–Munzel test (nonparametric, robust to unequal variances), again applying BH-FDR across windows and the same ≥6-bin criterion; results were consistent with the KS-based analysis.

#### SSs population discrimination between SR-left and SR-right

We defined a grid of thresholds in sliding 100 ms windows advanced in 50 ms step over two epochs: 100 ms before to 300 ms after the stimulus and from 300 ms before to 400 ms after the onset of the movement. At each threshold *t*, and for each condition, we counted how many neurons had already discriminated between the two associations versus those that had not yet discriminated. For every pairwise condition comparison (OT vs EL correct, OT vs LL correct, EL vs LL correct; and EL-correct vs EL-error), these counts formed a 2 × 2 contingency table (responded/no responded) which we tested with Pearson's *χ*^2^ (df = 1) to obtain a raw *p* value at each threshold. Because many overlapping thresholds are tested, raw *p* values were adjusted within each pairwise comparison across all thresholds using Benjamini–Hochberg FDR (*q* < 0.05). For each comparison, we recorded two onset times: (1) the earliest threshold with raw *χ*^2^ *p* < 0.05 in ≥2 consecutive thresholds and (2) the earliest threshold with FDR-adjusted *p* < 0.05 in ≥2 consecutive thresholds. To verify that any detected divergence was unlikely by chance, we performed a label-shuffle test. For each pairwise comparison, we pooled all latencies (including nonresponders), randomly reassigned condition labels while preserving group sizes, and repeated the entire sweep. For each of 1,000 permutations, we recorded the minimum *p* across thresholds and the first threshold at which *p* < 0.05. Comparing the observed values to the permutation distributions yielded empirical *p* values for (1) the global minimum *p* and (2) the first-cross time; a result was considered permutation significant if <5% of shuffles were as or more extreme.

### Quantification and statistical analyses of complex spikes

#### Single neuron’s CSs peak detection

For each neuron, we computed SDFs for single trials in two windows: (1) stimulus-aligned (0–300 ms after stimulus onset) and (2) movement-aligned (−200 to +200 ms around movement onset), analyzed separately for SR_left and SR_right with ≥5 correct trials. Within each window, we averaged the SDFs across trials to obtain a mean SDF and identified local maxima using the Matlab function findpeaks. To assess whether each peak reflected a genuine event-locked increase, we used a permutation test that preserved within-trial temporal structure while disrupting alignment to the event. Each trial's SDF segment was circularly shifted by a random offset within the window, and a permuted mean SDF was recomputed. Repeating this 1,000 times produced a null distribution of mean SDF amplitudes at every time point. For each observed peak, we computed a one-sided empirical *p* value as the fraction of permuted amplitudes ≥ the observed value. Peaks with *p* ≤ 0.05 were deemed significant, with Benjamini–Hochberg FDR applied when multiple peaks occurred.

#### Population-level complex spike probability analysis

We included neurons that showed at least one significant CS peak in either or both SR associations. If a neuron showed a peak only for one association, only those trials were used. For each neuron, we computed the per-ms mean spike count across trials and aggregated these into 20 ms sliding windows (1 ms step). Short windows were necessary given the low CS firing rate. Each window's expected spike count was converted to the probability of observing ≥1 spike using the Poisson approximation $P(\geq 1)=1-e^{-\lambda }$. This normalized responses to a 0–1 scale and increased sensitivity to small condition-dependent differences. We compared OT versus EL, OT versus LL, and LL versus EL using two-sided Wilcoxon rank-sum tests in each sliding window (*p* < 0.05). Significant effects had to persist for ≥20 ms. Within each run of significant bins, we applied a permutation test (1,000 shuffles) on the Wilcoxon statistic with Benjamini–Hochberg FDR correction; only FDR < 0.05 windows were retained. Gaps ≤8 ms were bridged to form continuous intervals, which were then retested at the interval level to ensure temporal and statistical robustness.

#### Simple spikes complex spikes correlation

To test whether modulation of CS corresponded to relative modulation of SS, we computed a correlation across neurons between per-neuron CSs and SSs in 100 ms consecutive bins, stepped every 2 ms. For each neuron and bin, we computed the probability of observing ≥1 spike using the Poisson approximation $P(\geq 1)=1-e^{-\lambda }$. We then normalized per neurons SSs firing rate by its 95th percentile. Across neurons, the CS probability and the normalized SSs were related in each bin using Kendall’s rank correlation test. Two-sided permutation tests (2,000 shuffles) were run per bin by shuffling SSs across neurons within that bin to form a null for (tau). We considered the *p* value as the fraction of permuted effects whose absolute value matched or exceeded the observed one. We required at least four consecutive bins of *p* < 0.05.

#### Neurons and trials selection, pseudotrials definition, and decoding of neuronal population activity

We analyzed the neuronal activity recorded in the overtrained (OT), novel (NT), and reversal (REV) tasks. During NT and REV tasks, we defined each session’s early and late learning phases as those trials with an average performance below and above 80%, respectively. We subsequently analyzed the neuronal activity of correct trials in early and late learning separately. In particular, we analyzed those sessions with at least five trials per condition, i.e., right and left responses, in correct and error trials. We analyzed error trials only in early learning because there were not enough trials per condition during the late learning phase.

Linear decoders were trained and tested on pseudosimultaneous population trials (pseudotrials). We defined a pseudotrial as the combination of spike counts randomly sampled from every neuron in a specific time bin and task condition (Rigotti et al., 2013). Pseudotrials were generated as follows: given a time bin *t* and task condition *p*, for every neuron, we randomly picked a trial of task condition *p*, and we computed the spike count in the time bin *t*. The single pseudotrial *ξ*, for condition *p* at time bin *t*, is then *p*(*t*) = (*ξ*_1_*^p^*(*t*), *ξ*_2_*^p^* (*t*),…, *ξ_Ν_^p^* (*t*)), where *N* is the number of recorded neurons and *ξ_i_^p^* (*i* is the neuron identity, *i* = 1,…,*N*) is the spike count. We repeated this procedure 100 times, with 1,000 pseudotrials per task condition and time bin. To disambiguate the visual stimulus from the response signals, we analyzed correct trials in the NT and REV tasks together, in the early and late phase, separately.

For each binary variable, i.e., stimulus and response, we trained a support vector machine (SVM) classifier with a linear kernel to classify the spike count into either of the two values of the binary variables—stimulus 1–2, left–right (Bishop, 2006; Pedregosa et al., 2011). The linear classifier was trained on pseudotrials built from randomly selected trials. In more detail, for every neuron, we selected 80% of the trials as a training set and the remaining 20% as a testing set to build the pseudotrials. We generated 1,000 pseudotrials for training and 1,000 pseudotrials for testing per condition. We randomly chose 80% of the training pseudotrials and 20% of the testing pseudotrials to train and test the linear decoder for a total of 100 cross-validations. We showed the final accuracy of the linear decoder as the ratio between the number of correct predictions and the total number of predictions on the testing set averaged across the cross-validations. To evaluate the statistical significance of the neural signal, we built a null model by randomly shuffling the task condition labels among the pseudotrials. We trained a linear decoder on the shuffled training set for each shuffle and assessed its accuracy on the shuffled testing set. We repeated the shuffle procedure 100 times, obtaining a null model distribution. We defined the chance interval as the interval between 2 standard deviations of the null model distribution around the chance level at 50%. We applied the same procedure above to decode both the neural and artificial data. For the neural data, we decoded the activity in a 200 ms time bin stepped by 20 ms along time from 200 ms before the stimulus onset until 800 ms after. We also decoded the neuronal activity from 400 ms before the movement onset until 800 ms after the response. All decoding analyses were performed using scripts of the scikit-learn SVC package (Pedregosa et al., 2011) and custom Python scripts.

#### Model of PCs input signals

In our first model, we modeled the visual and motor input signals to the PCs differently in early and late learning. During early learning, we modeled the visual input signals for stimuli 1 and 2 as a matrix *V*_1,2_ of (1,000 trials, 50 units, 150 time bins) dimensions. At the time bin 0, we set the stimulus onset, and it remains active for all following 150 time bins. Each element of the matrix *V*_1,2_ is defined as follows:

$$v_{i}^{1,2}=N(\mu^{1,2},\sigma^{1,2}),\;(1)$$

where i=1,…,50 indicates the unit ID; *N* is the Gaussian distribution with mean $\mu^{1,2}$ equal to −0.2 and −0.4 for stimulus 1 and stimulus 2, respectively; and a standard deviation $\sigma^{1,2}$ equals to 0.3 for both stimuli. The negative mean for visual input distribution simulates subthreshold signals that require learning-dependent amplification to drive PC activity, consistent with the delayed visual decoding observed in EL*.* For each time step and trial, we randomly sample a random value from the previous distributions for each unit.

We modeled the motor signals, i.e., response 1 and 2, as a matrix *M*_1,2_ of (1,000 trials, 50 units, 150 time bins) dimension. The movement onset was set at bin 100. Each element *m*_1,2_ of matrices *M*_1,2_ before and after the movement onset is defined as follows:

$$\;\mathrm{before}:\;m_{i}^{1,2}=N(\mu_{1,2}^{\mathrm{pre}},\sigma^{1,2}),\;\;\mathrm{after}:\;m_{i}^{1,2}=N(\mu_{1,2}^{\mathrm{post}},\sigma^{1,2}),\;\;(2)$$

where i=1,⋯,50 indicates the unit ID; *N* is the Gaussian distribution with mean $\mu_{1,2}^{\mathrm{pre}}$ equal to 0 for before the movement onset; and mean $\mu_{1,2}^{\mathrm{post}}$ equals to 0.2 and 0.4 for response 1 and response 2 after the movement onset, respectively. The standard deviation $\sigma^{1,2}$ is set equal to 0.3. In line with the behavioral task, we define two task conditions where visual stimuli 1 (S1) and 2 (S2) are univocally associated with motor responses 1 (R1) and 2 (R2), respectively. When the task condition, for instance, is S1-R1, all the units encoding by construction S2 and R2 are set to NaN during the simulation. We defined a total input matrix *I*^input^ of (1,000, 200, 150) dimension obtained by concatenating *V*_1,2_ and *M*_1,2_. This input matrix is passed through 100 rectified linear units (ReLU) whose weights *W* are randomly sampled from a uniform distribution *U*(0,1). The final activity output readout by a linear decoder at time bin *t* is as follows:

$$u(t)=[WI^{\mathrm{input}}(t){]}_{+},\;(3)$$

where $I^{\mathrm{input}}$ is the input matrix and $\left[x{]}_{+}=\;\max(0,x\right)$ is the ReLU nonlinear function mapping the input currents to positive firing rates.

#### Training of an artificial neural network to perform the visuomotor association task

We trained an artificial feedforward neural network to perform the NT and REV tasks. We provided a five-unit input to the network where a one-hot vector of three units encodes the context: the stimulus-only context and task context, i.e., NT or REV tasks. The last one-hot vector of two units encodes the stimulus identity. The stimulus identity is always provided to the network along with one of the three contexts. The stimulus-only context requires the network to provide the stimulus identity as output regardless of the response (which is random); the task context requires the network still to provide the stimulus identity and the response associated with it according to the NT or REV task.

We introduced the stimulus-only context to reproduce the neural signal activity where the visual stimulus is decoded earlier than the response in the late learning phase. In particular, we wanted to model the activity of the visual and response neural signals along the trial: in the first three artificial time points, the network receives the stimulus-only context as input to reproduce the onset of the stimulus signal during the experiment. Subsequently, the network receives the task-contextual input and the stimulus identity input, instructing the network on whether to perform the NT or REV task. The stimulus identity is always provided with different contextual inputs at different time points along the trial.

In each time point, the selected input *I* is processed by an expansion layer of 400 linear units through random expansion with initial weights *W*^in^ randomly sampled from a uniform distribution *U*(0,1). We refer to these units as artificial granule cells (aGCs). The *W*^in^ are fixed along the training. The output current *α* from the aGC unit *j* is defined as the weighted sum of the input as follows:

$$\alpha_{j}=\sum_{i=1}^{i=5}\;W_{\mathrm{ji}}^{\mathrm{in}}I_{i},\;(4)$$

where *i* is the input unit index. The output current is passed to a layer of 100 ReLU units through *W*^out^ weights randomly initialized from a uniform distribution *U*(0,1), which will be trained. We refer to these units as artificial Purkinje cells (aPCs). The output current *β* of an aPCs unit *k* is defined as follows:

$$\beta_{k}={\left[\sum_{\;j=1}^{\;j=400}\;W_{\mathrm{kj}}^{\mathrm{out}}\alpha_{j}-b\right]}_{+},\;(5)$$

where *j* is the index of an aGC input unit, $\left[x{]}_{+}=\;\max(0,x\right)$ is the nonlinear function (ReLU), and *b* = 15 is the bias term, and it remains fixed along the training. The output current from aPCs layers is read by two output units *R*,*S*, which give the stimulus ID and the associated response. The two readouts are defined as follows:

$$\begin{array}{rl} & S=\mathrm{sgn}[B^{\prime }-\theta ],B^{\prime }=\sum_{k=1}^{k=60}\;\beta_{k};\;\;R=\mathrm{sgn}[B^{\prime \prime }-\theta ],\\ & B^{\prime \prime }=\sum_{k=41}^{k=100}\;\beta_{k}, \end{array}\;\;\;\;\;(6)$$

where θ=1 and $B^{\prime }$ and $B^{\prime \prime }$ are the total output currents from the preceding layer’s first 60 and last 60 aPCs units. In other words, the two output units *S* and *R* read out the activity from two different pools of aPCs units with an overlap of 20 units, i.e., 20%.

The network training is performed over a total of 1,000 training trials. In the first 500 trials, the network is trained to receive the stimulus-only and NT-task contexts as input randomly chosen for each trial. In the second half of the training trials, the network is trained to receive the stimulus-only and REV-task contexts. After switching from the first half to the second training phase, we reinitialized the output weights *W*^out^ of the last 5% units readout by the stimulus *S* output unit. This is one difference from the actual experiment because we force the network to relearn the stimulus identity input in order to disambiguate it from the response signal in the following decoding analyses.

The *W*^out^ weights are updated in each training trial *t* according to the following rule:

$$\delta W_{i}^{\mathrm{out}}=(R_{t}-R_{t}^{*})\times \mathrm{aG}C_{i},\;(7)$$

where $\mathrm{aG}C_{i}$ is the output current from the *i*-th aGC unit; *R*_t_ is the response the network provides at trial *t*, i.e., ±1; and $R_{t}^{*}$ is the correct response. The same learning rule is applied to the weights read out by the Stimulus ID unit *S*. The new output weight is as follows:

$$W_{i}^{\mathrm{outnew}}=W_{i}^{\mathrm{out}}+\lambda \delta W_{i}^{\mathrm{out}},\;(8)$$

where λ is the learning rate, which is set equal to 0.1 and remains constant throughout the training. The network's performance in providing the correct stimulus identity and the correct response is tested on a batch of newly generated 100 trials before every training trial. A small amount of noise randomly sampled from a Gaussian distribution of zero mean and variance equal to $10^{-3}$ is added to the input.

## Results

The current study is a reanalysis of previously published data (Sendhilnathan et al., 2020; Sendhilnathan et al., 2021). Two monkeys were trained for several months to perform a visuomotor association task where they had to associate one visual stimulus with a left-hand movement and another visual stimulus with a right-hand movement.

### Task

The monkeys began each trial by placing their left and right hand on a bar on the left and right side, respectively (Fig. 1*A*), after which a white square, the fixation point, appeared at the center of the screen. Monkeys had to fixate on the white square for an interval between 800 and 1,000 ms, and then one of two stimuli appeared for 200 ms instructing them to release the left hand or the right hand. Monkeys had 3 s to release the bars starting from the onset of the stimulus. We trained the monkeys on the overtrained task (OT) with the same pair of stimuli: a green square required the release of the left hand and a pink stimulus the release of the right hand until they reached performance accuracy close to 100%. We began each experiment with 20–30 trials using the overtrained stimuli, making sure that the monkeys performed with a success rate of at least 90%. We then switched the stimuli to fractal pictures that the monkeys had never seen before (new task, NT). Once behavioral performance reached ∼80% correct responses, the stimulus–response mappings were reversed (REV), requiring the monkey to acquire the new associations (Fig. 1). Because of the structure of OT and NT tasks, the stimuli—stimulus 1 and stimulus 2—and responses, right and left, are perfectly correlated; trials were categorized according to the required bar release: stimulus–response (SR)-left trials, in which the correct response was a left-bar release, and SR-right trials in which the correct response was a right-bar release. All subsequent analyses of “SR-left” or “SR-right” activity refer to these trial types.

**Figure 1.**
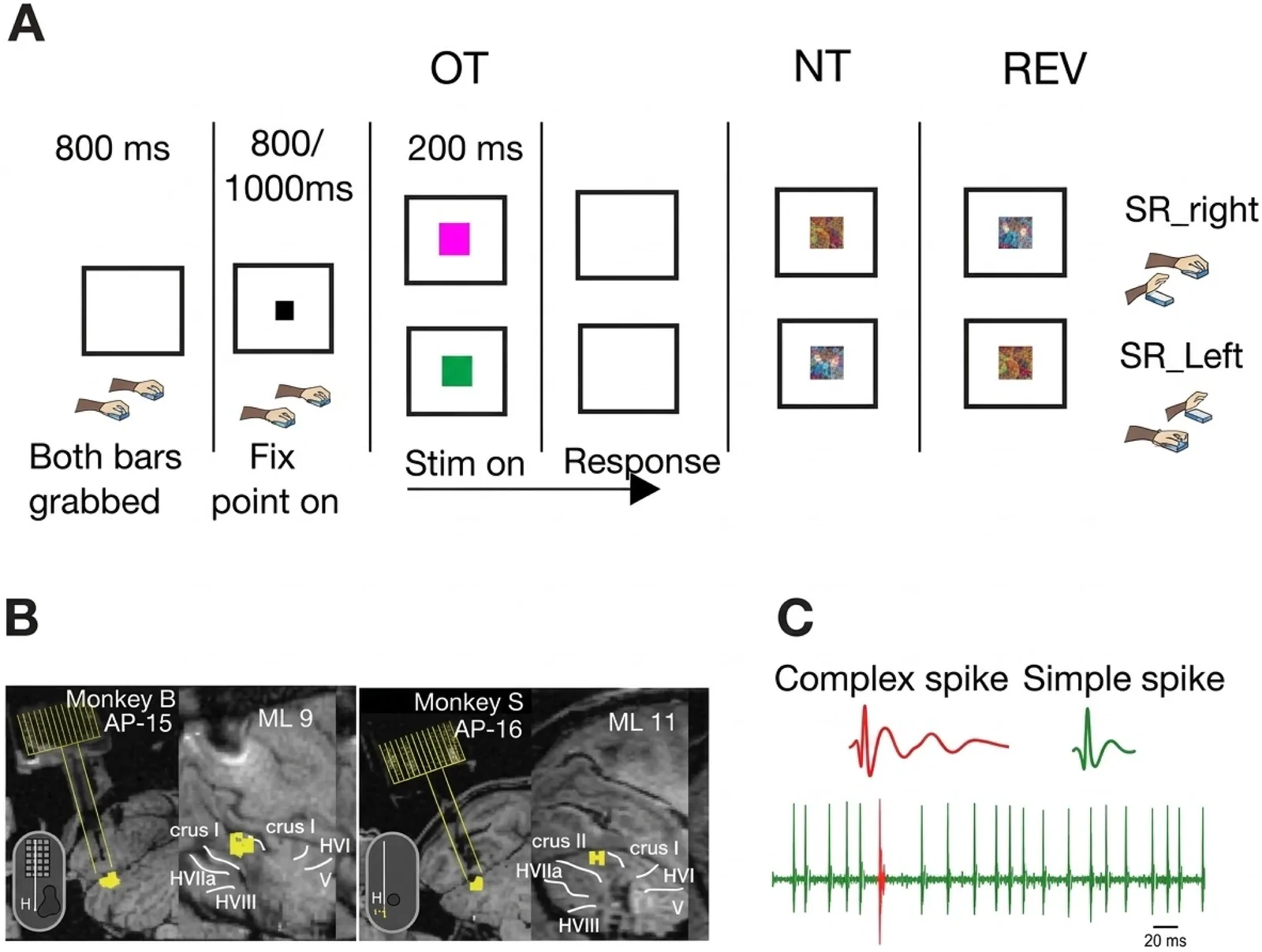
Behavioral task and recording locations in Crus I and Crus II. ***A***, Visuomotor association task. Each session began with the overtrained task (OT). Trials started when the monkey grasped two bars with the left and right hands. After the monkey maintained this position for 800 ms, a fixation point appeared. Following an additional variable delay of 800–1,000 ms, a visual stimulus was presented for 200 ms. In the OT condition, the green stimulus instructed release of the left bar (SR-left), whereas the pink stimulus instructed release of the right bar (SR-right). Monkeys had up to 3 s from stimulus onset to respond. The OT block was followed by a new task (NT), in which two novel stimuli, never previously seen by the monkey, were introduced. After performance reached ∼80% correct, the stimulus–response associations were reversed (REV), requiring the monkey to learn the new associations. Across task conditions, stimuli shown in the top row instructed right-hand release, whereas stimuli shown in the bottom row instructed left-hand release. SR-left, stimulus–response association requiring left-hand release; SR-right, stimulus–response association requiring right-hand release; OT, overtrained task; NT, new task; REV, reversal. ***B***, Recording locations. T1-weighted MRI images show the reconstructed recording locations in Monkey B and Monkey S. Yellow markers indicate recording sites. For each monkey, the left panel shows the coronal slice containing the recording chamber and burr-hole tract, whereas the right panel shows the sagittal reconstruction of the recording locations in Crus I and Crus II. ***C***, Representative Purkinje cell recording. Example raw extracellular recording showing simple spikes and complex spikes from a Purkinje cell. Simple spikes are shown in green, and complex spikes are shown in red.

### Behavior

Monkeys typically transitioned from a mix of correct and incorrect responses to near-perfect performance in an almost single-step fashion. Because accuracy computed in fixed trial bins would blur this abrupt shift, we instead fit a binomial logistic regression to trial-by-trial correctness to estimate the learning curve and pinpoint the inflection point. Trials with predicted probability (correct) <0.8 were classified as early learning (EL), whereas those with probability ≥0.8 were classified as late learning (LL).

Figure 2*A* illustrates the progression from chance-level to proficient performance in four representative sessions (two each from Monkeys B and S). The left panels show trial-by-trial responses (red, right-hand release; blue, left-hand release), with correct trials plotted at the top and incorrect at the bottom. The black curve is the binomial logistic regression fit, and the vertical dotted line marks the first trial where the predicted probability of correctness exceeded 0.80. The right panels show the number of correct and incorrect releases for each hand before this 0.80 threshold.

**Figure 2.**
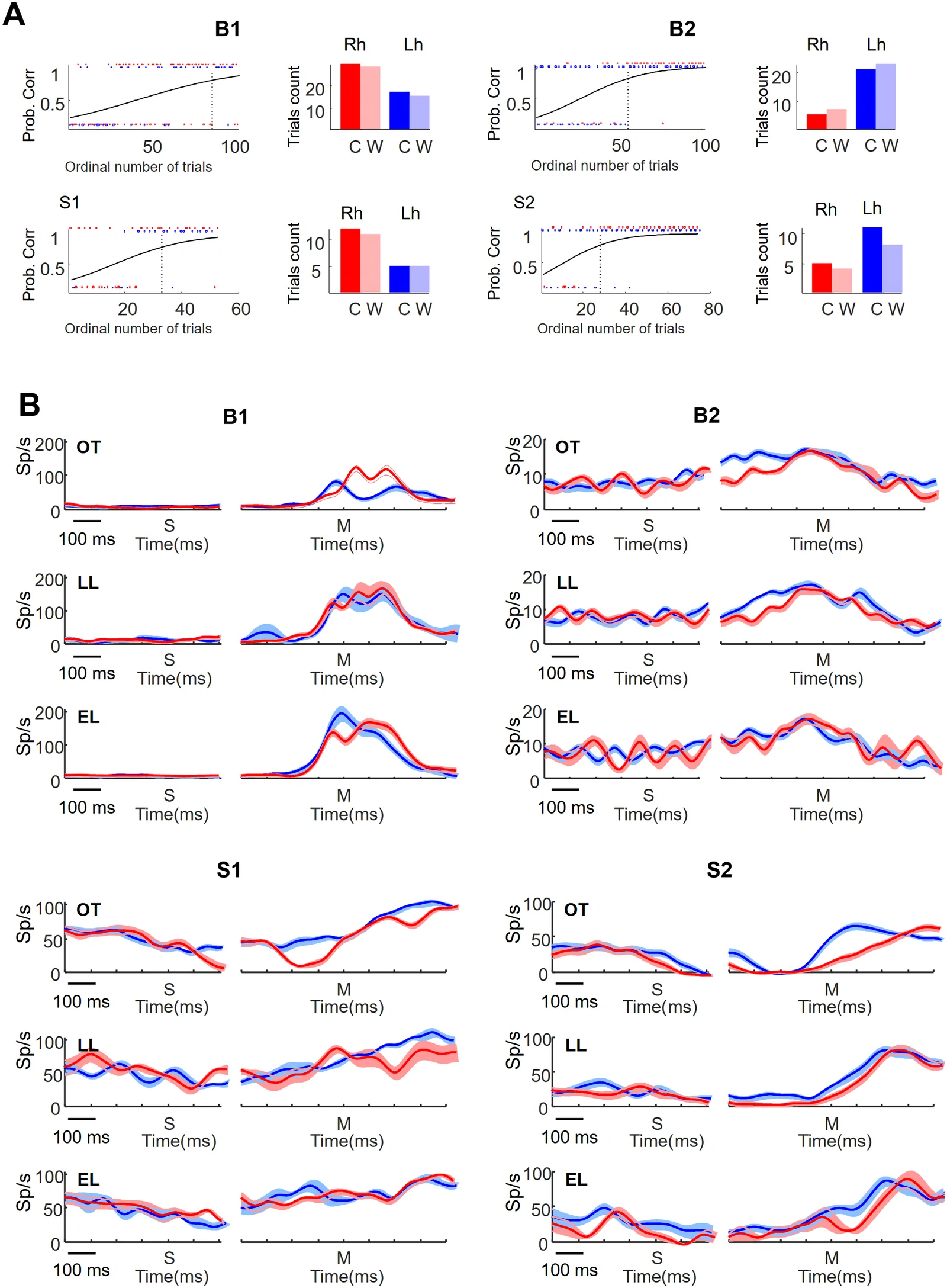
Four representative sessions from two neurons in Monkey B (B1, B2) and two in Monkey S (S1, S2). ***A***, Behavioral performance during acquisition of the new task. Left panels, Trial-by-trial performance. *x*-axis, ordinal trial number; *y*-axis: probability of a correct response. Solid line, binomial logistic regression fit of correctness probability. Symbols, top, correct trials; bottom, error trials; red, right-hand release; blue, left-hand release. Right panels, Absolute number of trials in the early learning stage (EL), showing correct (bright colors) and incorrect (pale colors) hand releases (red, right hand; blue, left hand). ***B***, Firing rate modulations during correct stimulus–response associations for SR-left trials (blue) and SR-right trials (red) across overtrained (OT), late learning (LL), and early learning (EL) conditions. Spike-density functions were computed using a Gaussian kernel with *σ*  =  15 ms. Traces are aligned to stimulus onset (S) and movement onset (M). The *x*-axis indicates time (ms), and the *y*-axis indicates firing rate (sp/s: spikes per second). Shaded areas indicate SEM.

We observed two key features of behavior before and during learning: (1) the number of trials required to reach a stable performance level (defined as ≥0.8 probability of a correct response) varied markedly across sessions (from ∼20 to ∼70 trials), and (2) monkeys frequently perseverated on one hand during early learning, creating highly imbalanced per-hand trial counts. For example, in Session B1 (Fig. 2*A*, left panel), Monkey B reached the threshold at ∼80 trials, with only 10 correct EL trials for SR-left versus 30 for SR-right, while in Session B2 (Fig. 2*A*, right panel), the threshold was reached at ∼50 trials, with 20 correct SR-left trials but only five correct SR-right trials. We observed a similar imbalance for Monkey S (Fig. 2*A*, sessions S1, S2). Because of such imbalances, the EL phase could contain far fewer correct trials for one hand. For all neuronal analyses, we therefore required a minimum of five correct EL trials per condition (left-hand and right-hand release) as an inclusion criterion. This ensured that both conditions were adequately represented despite session-specific perseveration patterns.

### Analysis of neuronal recordings

We recorded the activity of SSs in 80 Purkinje cells (PCs) in the right posterolateral cerebellum, predominantly in Crus I and II in two monkeys (Fig. 1*B*). We only analyzed cells that were stable throughout the entire session, showing a clean climbing-fiber pause. We only included units whose spike waveform shape and amplitude remained constant between the first and last trials and whose signal-to-noise ratio stayed high from start to end of each session (Fig. 1*C*). If a neuron's waveform or signal-to-noise ratio criteria were violated during recording, we halted data collection. In sessions that ended prematurely—because the monkey had not yet learned the association—those neurons were retained only for the “early learning” analyses.

### Neural activity modulation from baseline to stimulus–response interval

We next examined SS responses of individual PCs during correct SR-left and SR-right trials across learning stages. Figure 2*B* shows firing-rate profiles from stimulus onset through movement for four example neurons (B1, B2 from Monkey B; S1, S2 from Monkey S), plotted separately for early learning (EL), late learning (LL), and overtrained (OT). In OT, all four cells exhibited a significant deviation from their prestimulus baseline in both SR-left (blue) and SR-right (red) trials, yet each did so with a unique time course. Neuron B1's activity rose sharply ∼150 ms before movement in both conditions, whereas B2's modulation began even earlier and ramped steadily into the response. Cell S1 showed a brief inhibitory transient in both SR-right and SR-left trials, but stronger in SR-right, followed by a slower excitatory buildup, while S2 displayed an early poststimulus inhibition in both associations, then a premovement increase on SR-left trials and a continuous ramp in SR-right trials that returned to baseline ∼300 ms after movement. Comparable heterogeneity persisted in LL and EL—cells still responded to both associations, but with diverse latencies and polarities. These patterns indicate that single PCs encode different elements of the stimulus–response transformation at distinct moments during the trial.

Because different neurons started to discharge at different times after the stimulus onset, and there were different firing patterns in SR-left and SR-right trials, analyzing neural responses within fixed time intervals would not capture the full extent of this encoding, since fixed intervals assume uniformity in neural responses. Figure 3*A* shows, for each single neuron, the absolute difference between the *z*-scores of the neuron's firing rate in consecutive 80 ms bins, against its 100 ms prestimulus baseline in SR-left and SR-right correct trials, OT, LL, and EL. Although individual neurons reached their maximal difference at distinct times, the population as a whole “tiles” the entire trial—from stimulus onset through movement and persisted in OT, EL and LL, ensuring a continuous representation of SR-left and SR-right across the trial.

**Figure 3.**
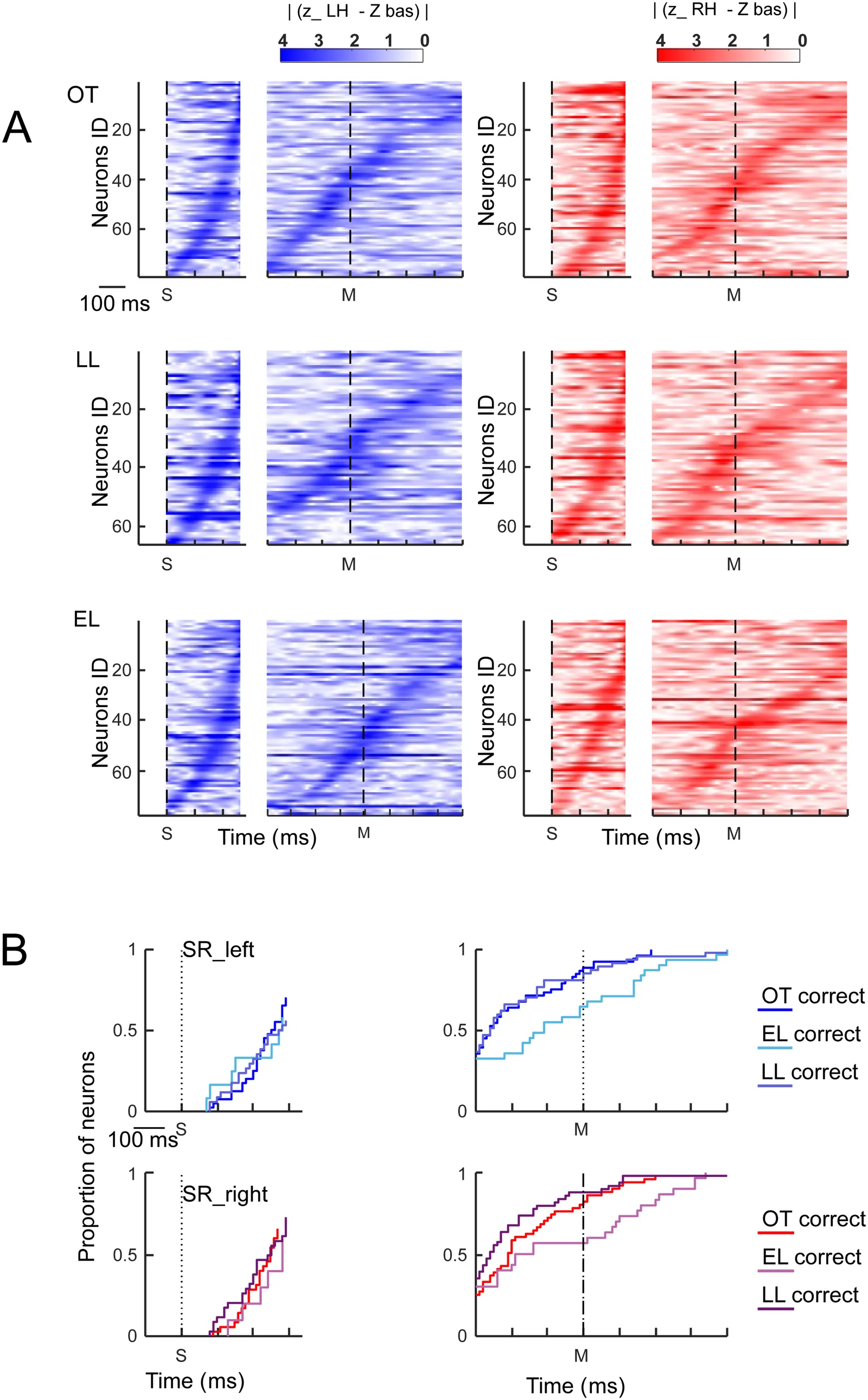
Neurons' responses to SR-left and SR-right correct trials. ***A***, Absolute *z*-scored firing rate differences from baseline for SR-left (blue) and SR-right (red) correct trials, aligned to stimulus (S) and movement (M) onset in overtrained (OT), late learning (LL), and early learning (EL) conditions. *y*-axis, neurons; *x*-axis, time (ms). ***B***, Cumulative proportion of neurons whose firing had already diverged from baseline by each time from the onset of the stimulus (S) and from the movement (M); *y*-axis, proportion of neurons; *x*-axis, times from the onset of the stimulus (S) and from the onset of the movement (M).

To capture the time course of neuronal response, for each neuron we compared the firing rate distributions in a sliding time window of 80 ms, stepped 10 ms, with the firing rate distribution computed during the baseline (defined as 100 ms time window before the onset of the stimulus). We defined the response time, the first of six consecutive bins, after stimulus onset, when the difference between the two distributions was significant (see Materials and Methods).

### Population-level emergence of significant responses from baseline

To examine how quickly neurons responded after baseline, we compared—across learning stages—the cumulative fraction of neurons that reached their individual response time (relative to baseline) at or before a series of cumulative cutoff thresholds. Thresholds were defined in 50 ms increments, meaning that the first threshold included all neurons responding within 50 ms, the second within 100 ms, and so on. This was done for two alignment windows: −100 to +300 ms relative to stimulus onset and −300 to +400 ms relative to movement onset. At each threshold, we calculated the proportion of neurons that had already responded significantly from baseline. Pairwise Pearson’s *χ*^2^ tests were applied to these proportions, with raw *p* values corrected via the Benjamini–Hochberg procedure and validated against chance using permutation-derived “minimum *p*” null distributions (see Materials and Methods). Figure 3*B* shows, for correct trials, the cumulative proportion of neurons that responded after baseline for SR-left trials (top) and SR-right trials (bottom), in the two alignment windows.

In the first 300 ms after stimulus onset, the proportion of responsive neurons did not differ across OT, LL, and EL in either SR-left or SR-right correct trials (Fig. 3*B*, left panels). In contrast, before movement onset, clear differences emerged at the −200 ms threshold. In SR-left trials, the proportion of responsive neurons in EL (11/56, 19.6%) was significantly lower than in OT (34/79, 43.0%; *χ*^2^ = 8.07, *p* = 0.0045, *ϕ* = 0.245) and in LL (31/66, 47.0%; *χ*^2^ = 10.02, *p* = 0.0015, *ϕ* = 0.287), with no difference between OT and LL. A similar pattern was observed in SR-right trials: the proportion in EL (13/59, 22.0%) was significantly lower than in OT (30/79, 38.0%; *χ*^2^ = 4.00, *p* = 0.0455, *ϕ* = 0.170) and in LL (34/66, 51.5%; *χ*^2^ = 11.54, *p* = 0.0007, *ϕ* = 0.304), again with no difference between OT and LL. Thus, while stimulus-aligned responses did not differ across learning stages, movement-aligned responses showed a clear enhancement in OT and LL relative to EL, suggesting that the timing of neural recruitment shifts earlier in the trial as associative learning progresses. In contrast, OT (*N* = 69) versus LL (*N* = 66) never reached two consecutive *χ*^2^ test *p* < 0.05. We did not find any significant difference in any pairs of comparison in the first 300 ms after the onset of the stimulus.

### Different Purkinje cells discriminate between stimulus–response left and stimulus–response right at different times

Although every cell responded in both SR-left and SR-right conditions in OT, most exhibited a clear difference in firing rates between the two (Fig. 2*B*). Figure 4 extends this comparison to the full population. Figure 4*A* presents heat maps of the absolute *z*-score differences between the firing rates SR-left and SR-right correct trials, calculated in 80 ms bins stepped every 10 ms (see Materials and Methods). Neurons are sorted by their maximal difference. In correct trials, this bin-by-bin SR-left versus SR-right comparison shows that neurons collectively “tile” the entire trial: as some cells reach their peak discrimination immediately after the stimulus, others do so closer to movement, and still others shift their peak across learning stages, ensuring that at every moment between stimulus onset and movement some subset of PCs maximally differentiating the two associations.

**Figure 4.**
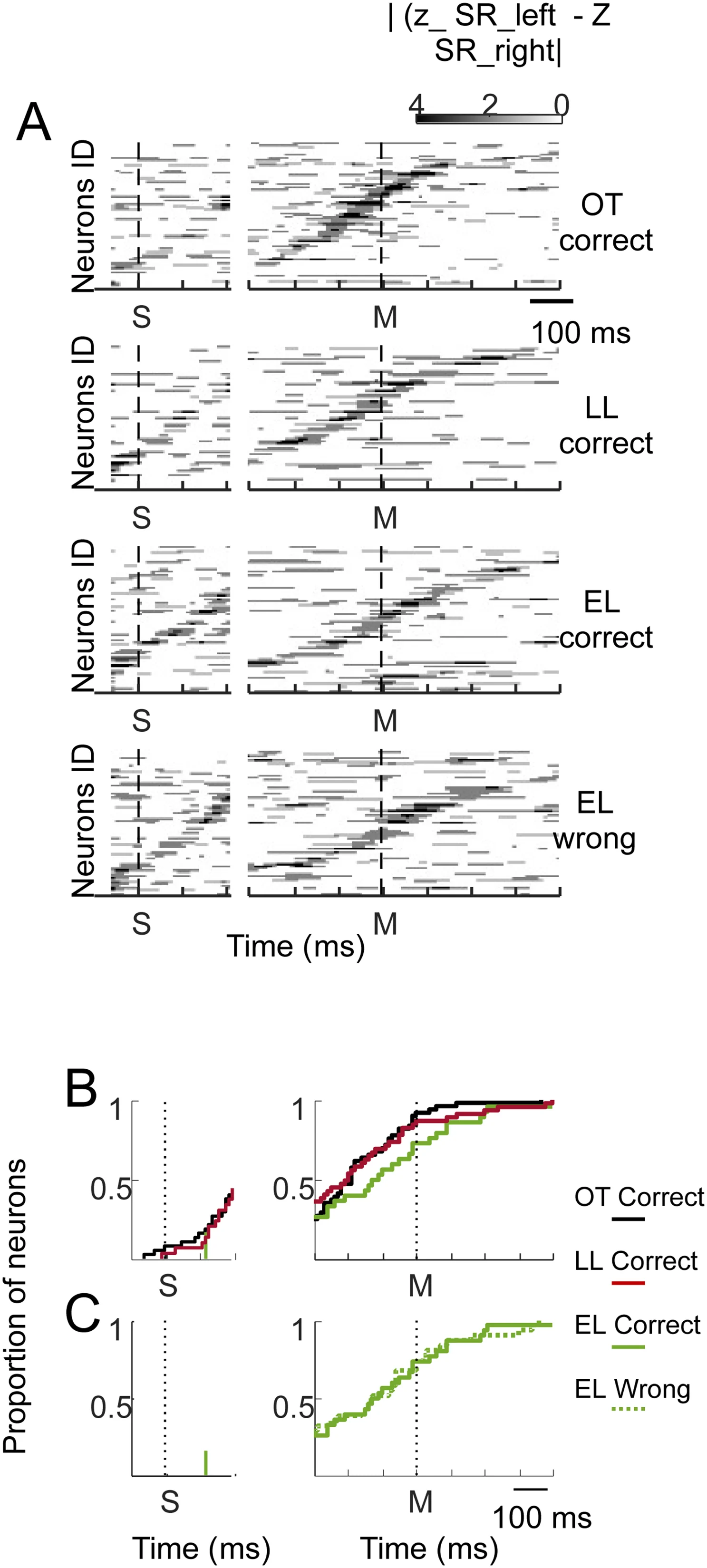
Purkinje cells discriminate between SR-left and SR-right at different times. ***A***, Heat maps of the absolute *z*-scored SR-left versus SR-right firing-rate absolute difference of the *z*-scores for correct trials in OT, LL, and EL. Values were computed bin-by-bin (80 ms bins, 10 ms steps) by directly comparing SR-left and SR-right firing rates (no baseline subtraction). *x*-axis, time (ms); the left subpanel is aligned to stimulus onset (S), the right subpanel to movement onset (M ). *y*-axis, neurons (one per row), sorted by the latency of their maximal absolute *z*-score difference. Vertical lines mark S and M onsets. ***B***, Cumulative proportion of neurons that had shown a significant SR-left versus SR-right difference by each time point (left, stimulus-aligned; right, movement-aligned). At every 10 ms threshold we built 2 × 2 responder/nonresponder tables for each stage pair and ran Pearson's *χ*^2^ tests, controlling across time with Benjamini–Hochberg FDR and validating significance with 1,000 label-shuffle permutations using the minimum-p metric (see Materials and Methods). ***C***, Same analysis as in ***B*** but comparing EL correct versus EL error trials.

To determine when individual neurons began to discriminate SR-right from SR-left associations, we measured firing rate distributions in an 80 ms sliding window stepped every 10 ms. At each window, we ran a two-sample Kolmogorov–Smirnov test on the two distributions and applied Benjamini–Hochberg correction across windows. Whenever the KS test reached significance, we performed a 1,000-iteration permutation test for validation. The discrimination latency was defined as the center of the first of at least six consecutive windows that survived both correction and permutation, ensuring that only sustained divergence was counted.

We characterized the population-level emergence of associative discrimination by comparing, for each learning stage, the cumulative fraction of neurons that had reached their individual discrimination time at or before each threshold. Here, a threshold refers to a cumulative cutoff time, stepped in 50 ms increments. For each candidate threshold (–100 to +300 ms around stimulus onset, and –300 to +400 ms around movement onset, in 50 ms steps), we calculated the proportion of neurons in each condition that had already discriminated between the two associations. Pairwise Pearson’s *χ*^2^ tests were then applied to these proportions, with raw *p* values corrected via the Benjamini–Hochberg procedure and validated against chance using permutation-derived “minimum *p*” null distributions (see Materials and Methods). Figure 4*B* (left) shows, for correct trials, the cumulative proportion of neurons that had already discriminated between the two associations in OT, EL, and LL, for thresholds from –100 to +300 ms after stimulus onset. Only a single neuron in EL reached the criterion during this period, and the proportion of discriminating neurons did not differ significantly between OT and LL. In contrast, at 250 ms before movement onset (Fig. 4*B*, right), the proportion of discriminating neurons was significantly higher in OT and LL compared with EL (OT vs EL: *χ*^2^_(1)_ = 4.5, *p* = 0.03, *ϕ* = 0.2; LL vs EL: *χ*^2^_(1)_ = 5.45, *p* = 0.01, *ϕ* = 0.2), with no difference between OT and LL. Because significant differences in discrimination timing were observed between EL and LL correct trials, even though the sensory stimuli and motor responses were identical, we hypothesized that these effects reflect ongoing acquisition of the stimulus–response mapping rather than differences in movement execution. Under this hypothesis, EL activity correct trials should resemble EL activity during error trials. To test this, we compared the proportion of neurons discriminating between left and right associations in correct versus error trials during EL. Figure 4*C* (left) shows that, within the first 300 ms after stimulus onset, only one neuron in correct trials and none in error trials reached the discrimination criterion. At movement-aligned thresholds (Fig. 4*C*, right), more neurons in both conditions had discriminated, but the proportions did not differ significantly at any threshold.

### Complex spikes

Complex spikes occur at low frequency and exhibit waveform variability over time, making reliable detection challenging. Although CSs were initially identified in all recorded PCs, we restricted our quantitative analyses to 26 neurons, in which spike sorting remained unambiguous throughout the session (see Materials and Method).

To determine whether complex spike (CS) probability differed across learning stages, we analyzed the per-neuron probability of at least one CS in 20 ms sliding windows, advanced in 1 ms steps, from stimulus onset and around movement onset (see Materials and Methods). Pairwise comparisons between learning stages (OT vs EL, EL vs LL, and OT vs LL) were performed using a two-stage procedure: first, a Wilcoxon rank-sum test was applied independently at each window; second, consecutive windows that reached significance in the pretest were evaluated with a permutation test. The permutation *p* value reflects the fraction of shuffled datasets in which the observed difference in mean CS probability could arise by chance. Intervals with *p* value < 0.05 were considered significant.

No significant differences in CS probability were detected between learning stages around movement onset. In contrast, clear learning stage differences emerged after stimulus onset, before movement initiation (Supplementary Fig. 1*A*). Visual inspection showed distinct temporal profiles in EL and OT. In EL, CS probability was relatively higher shortly after stimulus onset, whereas in OT it was initially lower and then increased at later poststimulus times. LL displayed an intermediate profile, with an early poststimulus peak followed by a decrease similar to EL and a later increase resembling the OT pattern.

Consistent with this temporal shift, OT showed a significantly lower CS probability than EL between 105 and 126 ms after stimulus onset (µOT − µEL = −0.048, *p* value = 0.03; *n* = 32 vs 20), followed by significantly higher CS probability at later intervals, from 144 to 166 ms (µOT − µEL = 0.032, *p* value = 0.02) and from 180 to 237 ms (µOT − µEL = 0.060, *p* value = 0.009). Compared with EL, LL showed significantly lower CS probability at 55–87 ms after stimulus onset (µLL − µEL = −0.040, *p* value = 0.01; *n* = 26 vs 20) and significantly higher CS probability at a later interval, from 191 to 218 ms (µLL − µEL = +0.047, *p* value = 0.01). No significant differences were detected between OT and LL at any time window, although the significant intervals observed in LL relative to EL were shorter than those observed for OT relative to EL.

We also compared CS probability between correct and error trials in EL (Supplementary Fig. 1*B*), where a sufficient number of error trials was available. CS probability did not differ significantly between correct and error trials at any time interval. Because of the low number of error trials in LL and OT, this comparison could not be performed reliably for those learning stages.

Finally, we examined the temporal relationship between CS occurrence and simple spike (SS) suppression (Supplementary Fig. 1*C*). In OT, CS probability was significantly and inversely correlated with SS firing rate from 111 to 250 ms after stimulus onset, indicating that higher CS probability was associated with lower SS activity. In contrast, no significant CS–SS correlation was detected in EL or LL. Thus, although the temporal profile of CS probability in LL began to resemble the OT pattern, the coordinated relationship between CS occurrence and SS suppression was evident only after the association was fully learned.

### Stimulus–response signal decoding

Given the learning-dependent shift in the CS responses we observed, we decided to further analyze activity of the SSs, which in the mouse behave reciprocally to the CSs (Gao et al., 2012; Badura et al., 2013). Thus, if the peak in CS activity shifts toward a later moment upon visual stimulation, one expects the SSs of the population of PCs to shift toward an earlier moment. In the following analyses, we trained SVMs (Bishop, 2006; Pedregosa et al., 2011) with a linear kernel to decode from the neural activity of PCs the future response after the stimulus onset and before the movement onset and the actual response after the movement onset. We analyzed correct and error trials separately. In this first analysis, we could not disentangle the response signal, i.e., right and left, from the stimulus identity signal, i.e., stimulus 1 and stimulus 2, and we will continue referring to this signal as to the stimulus–response (SR) signal. We trained and tested linear decoders on pseudosimultaneous trials (pseudotrials). We defined the pseudotrial as the combination of simple spike counts randomly sampled from different trials of the same task condition. We computed, for each neuron, the *z*-scored activity in a 200 ms time bin, stepped by 20 ms. In order to have enough training and testing trials for the linear decoders, we analyzed the activity of neurons recorded for at least five correct trials in each response condition (right and left) in OT, EL, and LL for a total of 54/80 neurons in OT, 18/80 neurons in EL, and 42/80 neurons in LL. The decoder identified the monkey's response after stimulus onset (Fig. 5*A*) and before movement onset (Fig. 5*B*) for correct trials. These results are qualitatively consistent across the two monkeys, with cleaner and more robust effects observed in one of the two monkeys (Supplementary Fig. 2). The average reaction times (mean ± SEM) in OT, EL, and LL, were 412 ± 4 ms, 760 ± 24 ms, and 519 ± 5 ms, respectively (Supplementary Fig. 3), confirming our prediction that the latencies of the simple spike responses were shorter following training.

**Figure 5.**
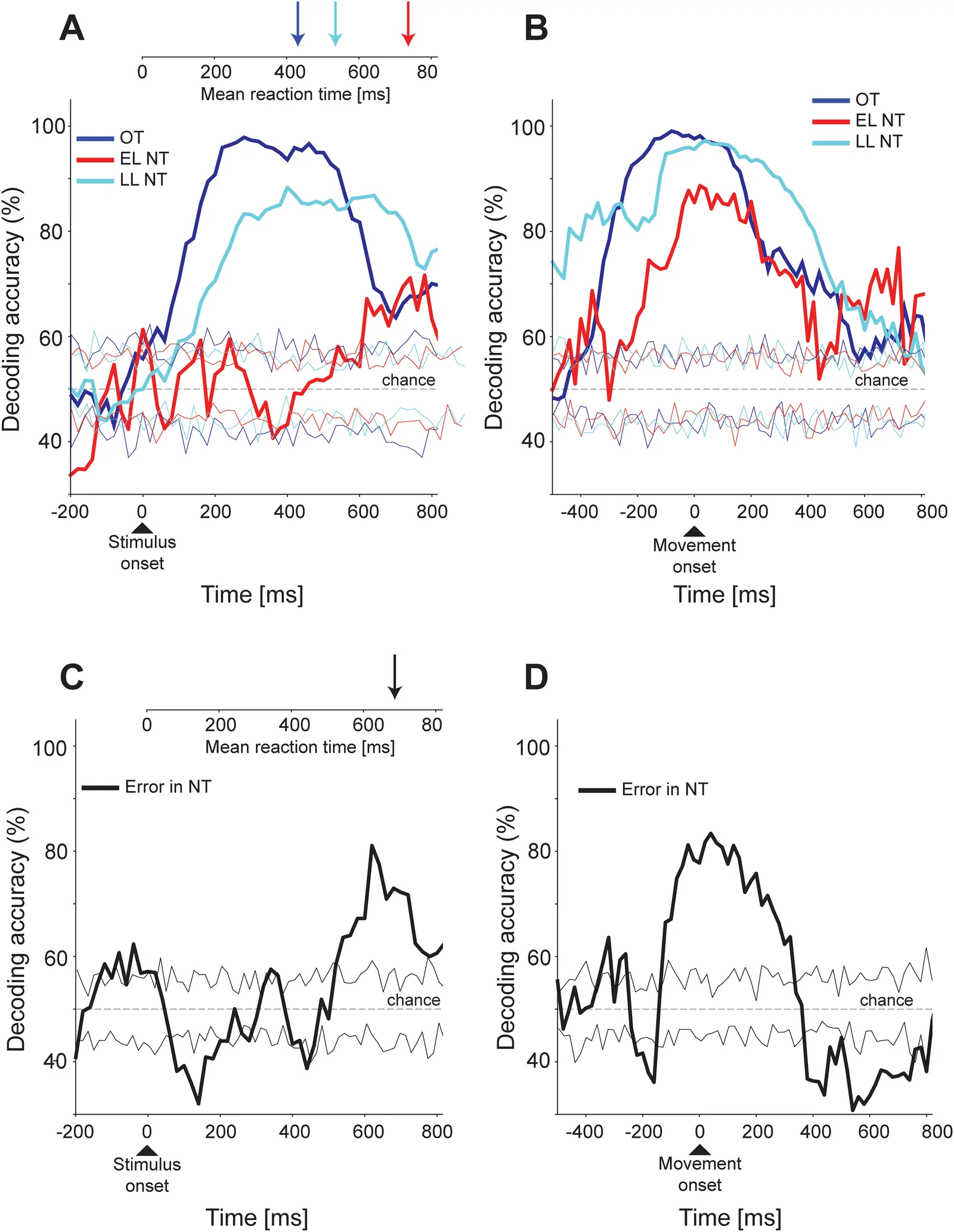
Population linear decoding of the stimulus response (SR) simple spike signals during overtrained task (OT), in early (EL) and late learning (LL) of novel task (NT). ***A***, Performance of a linear classifier in decoding the response around stimulus onset in OT (blue curve), in the EL (red curve), and LL (cyan curve) for correct trials. On top of the decoding accuracy, the vertical arrows indicate the mean reaction time. The horizontal dashed lines are ±2 standard deviations of the distribution of decoding accuracies from 100 null models with random shuffles of the labels for each time bin. ***B***, The same as in ***A*** but around the movement onset. ***C***, Performance of a linear classifier in decoding the response around stimulus in error trials during NT. On top of decoding accuracy, the vertical arrows indicate the mean reaction time. The horizontal dashed lines are ±2 standard deviations of the distribution of decoding accuracies from 100 null models with random shuffles of the labels for each time bin. ***D***, The same as in ***C*** around the movement onset.

We subsequently analyzed error trials during the novel task (NT), and we retained those sessions with at least five trials per condition, for a total of 24/80 neurons. The analysis of error trials showed that the SR is not decoded immediately after the stimulus onset. Instead, it occurs only after ∼600 ms, which is the average reaction time during error trials (Fig. 5*C*,*D*). The activity during error trials in EL was similar to the activity during correct trials in EL, where there was no significant signal after the stimulus onset (as seen in Fig. 5*A*, red curve). In error trials, around the movement onset, the signal became significant only at the moment of the response, but it dropped to chance in ∼300 ms after the response. This was different from the activity during correct trials in NT where the signals remained significant for ∼400 ms in EL and ∼600 ms in LL (this is true also for each single subject; Supplementary Fig. 2). The average reaction time in error trials was 691 ± 17 ms. We observed high variability in the reaction times across trials and across sessions, particularly in correct trials in EL and error trials (Supplementary Fig. 3).

We also decoded the outcome of the previous trials before the stimulus onset and the outcome of the current trial around the movement onset during early learning. We found that the previous outcome was significantly encoded in the 500 ms preceding the stimulus onset (Fig. 6*A*). The outcome of the current trial was significantly decoded ∼400 ms after the movement onset (Fig. 6*B*).

**Figure 6.**
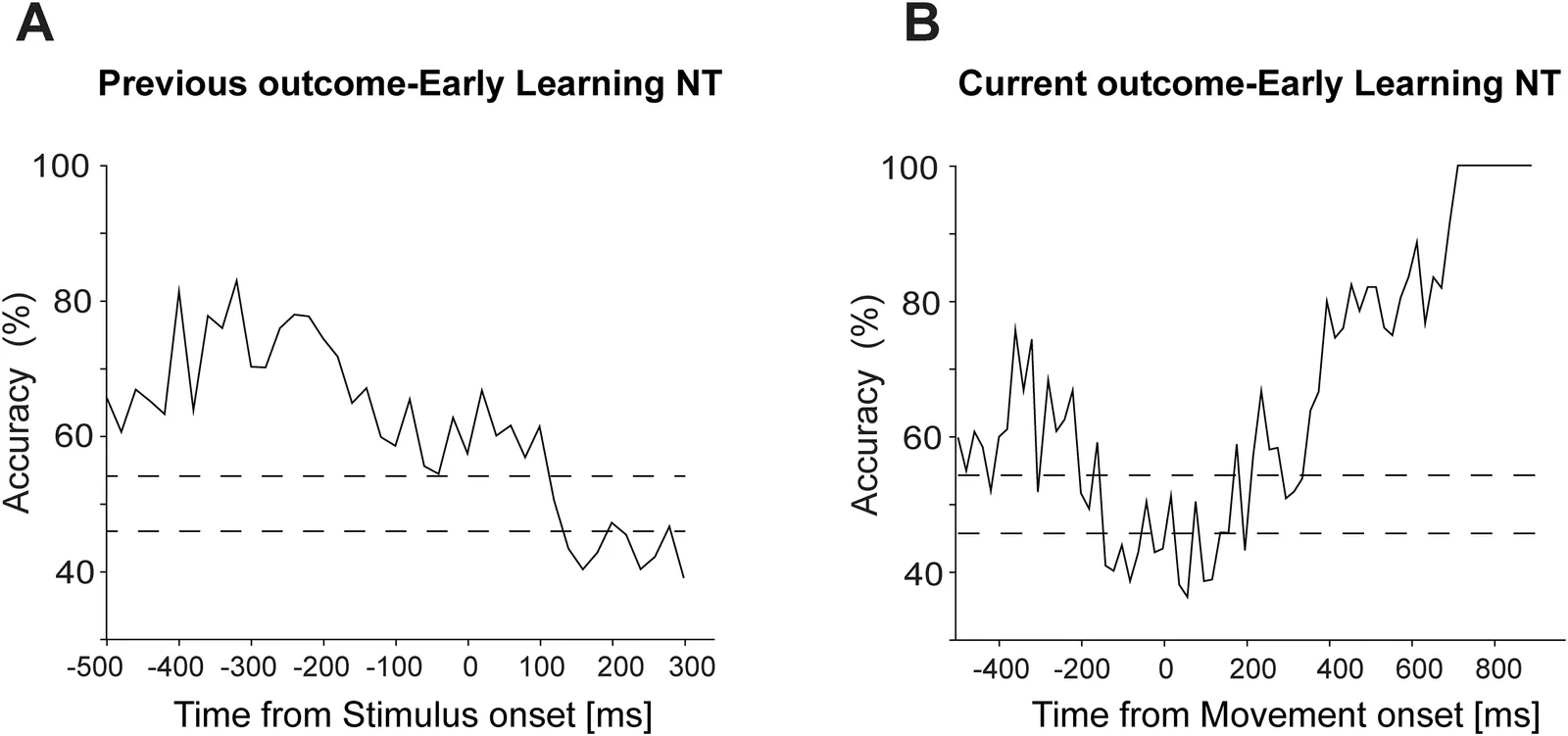
Decoding of previous and current outcome. ***A***, Decoding of the outcome of previous trials around current stimulus onset during early learning of novel task (NT). ***B***, Decoding of the current outcome around the movement onset during early learning of novel task (NT).

### Stimulus–response signal stability analysis

To test the stability of the signal over time, we used cross-bin decoding, which involved training a linear decoder in one time bin and testing it in a different time bin. If the decoder's accuracy in generalizing across time bins differs from the chance interval obtained from the distribution of accuracies of null models, it suggests that the variable’s representation is consistent over time. Figure 7 shows the cross-bin decoding accuracy of the linear decoder in classifying the response during OT and NT. For this analysis, we combined the data from EL and LL (see Supplementary Figs. 4 and 5 for the results separately for EL and LL). We found that the response representation is more stable during OT than NT after the stimulus onset (as shown in Fig. 7*A*,*B*). We defined the time interval during which the generalization accuracy is significant before it reaches chance level as the difference between the time bin used during training and the first testing time bin where the decoding accuracy is no longer significant. We only did the analysis in the forward direction, where the testing time bin is the same as or after the training time bin. After the stimulus onset, the time interval with a significant decoding accuracy is longer in OT (295 ± 34 ms, mean ± SD) than that in NT (154 ± 37 ms; Supplementary Fig. 4; Mann–Whitney *U* test; *p* value = 1.2^−11^). This finding suggests that after the stimulus onset, if the information is significantly encoded in both tasks, the response representation is less stable across time in NT than OT, indicating a faster reconfiguration of the decoding weights (Supplementary Fig. 6). Interestingly, after the movement onset, we observed that the signal is more stable across time in NT (187 ± 106 ms) than that in OT (140 ± 74 ms; Mann–Whitney *U* test; *p* value = 0.001).

**Figure 7.**
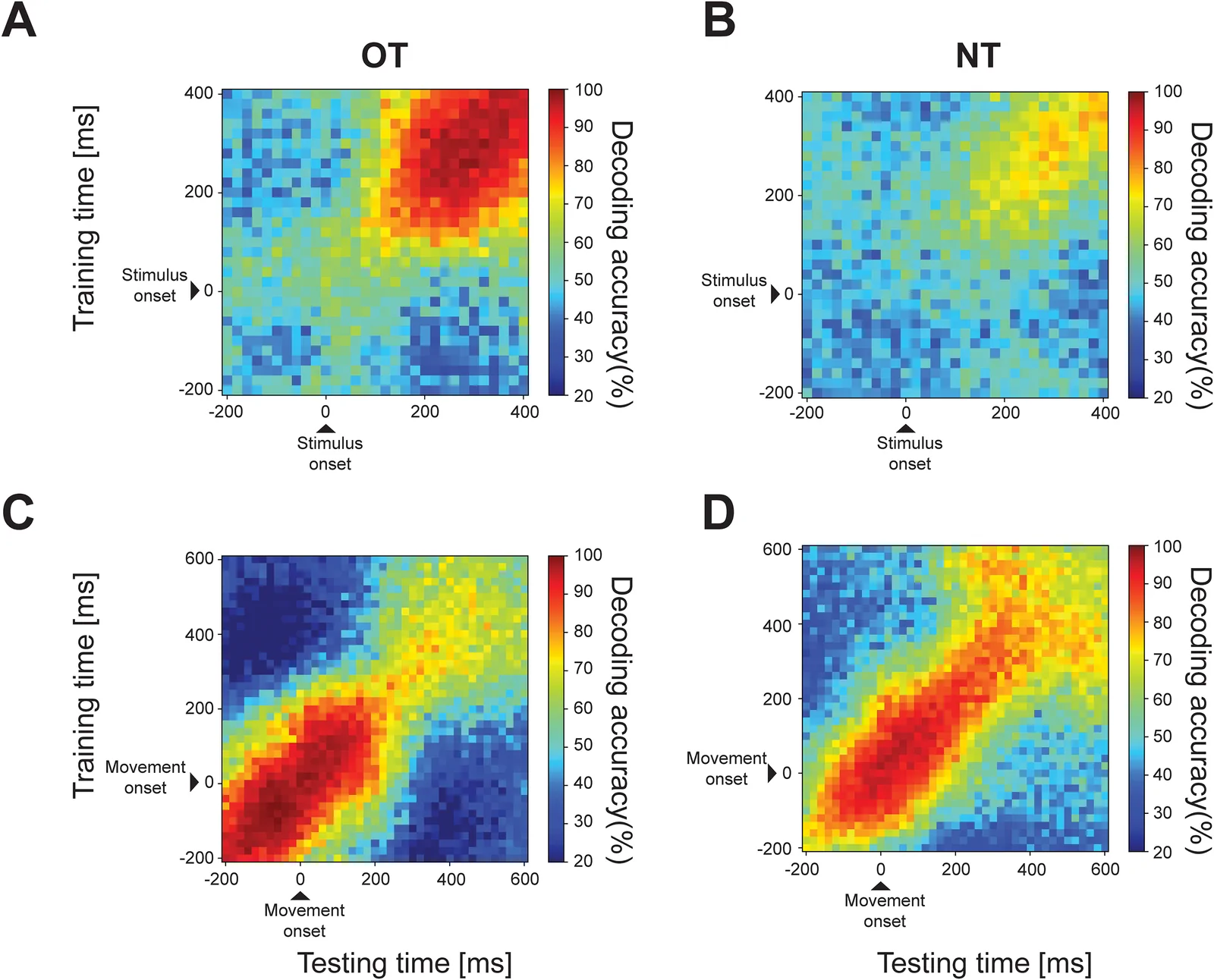
Cross-bin decoding in correct trials during overtrained (OT) and novel (NT) tasks. ***A***, Cross-bin decoding around stimulus onset during OT. The linear decoder is trained on a time bin (*y*-axis) and tested on other time bins (*x*-axis) preceding and following the training bin. The diagonal contains the decoding accuracy where the decoder is trained and tested on the same time bin. The color bar is the performance of the linear decoder in generalizing across time bins. ***B***, The same as in ***A*** during NT. ***C***, Cross-bin decoding around movement onset during OT. ***D***, Same as in ***C*** during NT.

### Neural decoding of the response and stimulus identity signals

To differentiate the amount of information encoded by the PCs for the simple spike response (right and left) from the visual signal (Stimulus 1 and Stimulus 2), we examined the correct trials of the novel and reversal tasks together. In the reversal task (REV), the visual stimulus and the response are reversed with respect to the novel task, making the response and stimulus identity uncorrelated. We defined the early and late learning phases as those trials in which the average behavioral performance in each session is between 40 and 60% and above 70%, respectively, in both novel and reversal tasks. We chose these thresholds to maximize the number of trials and neurons both in NT and REV tasks. During EL, the decoder could not identify the stimulus identity after the stimulus onset, except for a small bump after ∼200 ms from the stimulus onset (Fig. 8*A*). During LL, the decoder identifies stimulus identity after ∼100 ms from the stimulus onset, followed in time by the response signal (Fig. 8*C*). In EL, around the movement onset, the decoder identifies the stimulus identity before the movement onset (Fig. 8*B*). This signal is maintained after the movement onset for ∼200 ms, unlike in LL, where the stimulus identity signal drops to chance right after the movement onset (Fig. 8*D*).

**Figure 8.**
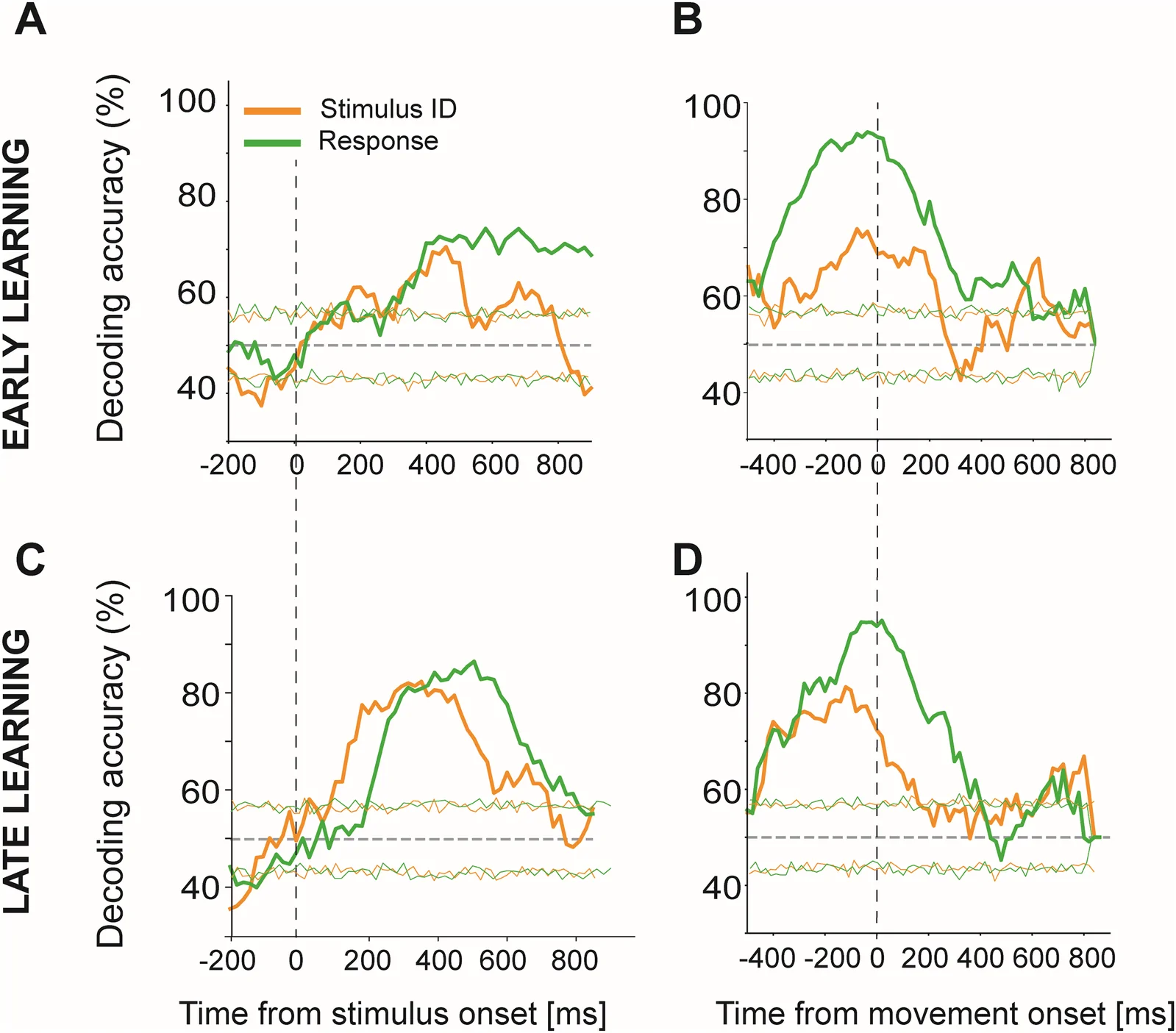
Decoding of the response and stimulus identity in the early and late learning phases of novel (NT) and reversal (REV) tasks using correct trials. ***A***, Stimulus identity (orange curve) and response (green curve) signal from the stimulus onset after combining the correct trials in the early learning of NT and REV task. ***B***, The same as in ***A*** around the movement onset. ***C***, Stimulus identity (orange curve) and response (green curve) signal after the stimulus onset after combining the correct trials in the late learning of NT and REV task. It is evident that the stimulus identity is decoded after ∼100 ms from the stimulus onset, followed in time by the response signal. ***D***, The same as in ***C*** around the movement onset. The horizontal dashed lines are 2 standard deviations of the distribution of decoding accuracies obtained from 100 shuffles of the labels for each time bin.

We also decoded the response and stimulus identity signals after combining the correct and error trials during EL and LL of novel task. Supplementary Figure 7*A*,*C* shows that, during early and late learning, there were no significant signals above chance in the 200 ms following the stimulus onset. However, during LL, the stimulus identity is decoded shortly after the stimulus onset, followed by the response signal (Figure 8*C*). This difference might be explained by the nature of the tasks being analyzed. Indeed, during the reversal task, the animals were not new to the stimulus identity, as they had previously been trained on the same stimuli in the novel task. In the reversal task, they were asked to reverse the stimulus–response association with respect to the association learned in the novel task. In contrast, during the novel task, the stimulus identities were entirely new to the animals, which is why there was no significant signal after stimulus onset in the early and late learning sessions (Supplementary Fig. 7*A,C*). This differs from what was observed when we analyzed correct trials in the novel and reversal tasks (Fig. 8*A*,*C*).

### Potential mechanism underlying different timing of EL and LL signals

To investigate the potential mechanism behind the different timing of stimulus and simple spike response signals in the EL and LL, we conducted two modeling studies aimed at reproducing the neuronal signals depicted in Figure 8. In the first model, we hypothesized that the visual input to the PCs became more specific during learning. In the second model, we trained a simple feedforward neural network to perform the NT and reversal (REV) tasks. After achieving high accuracy in learning the tasks, we analyzed the activation of the artificial units over time, employing the same analyses that we applied to real Purkinje cell activity.

**Figure 9.**
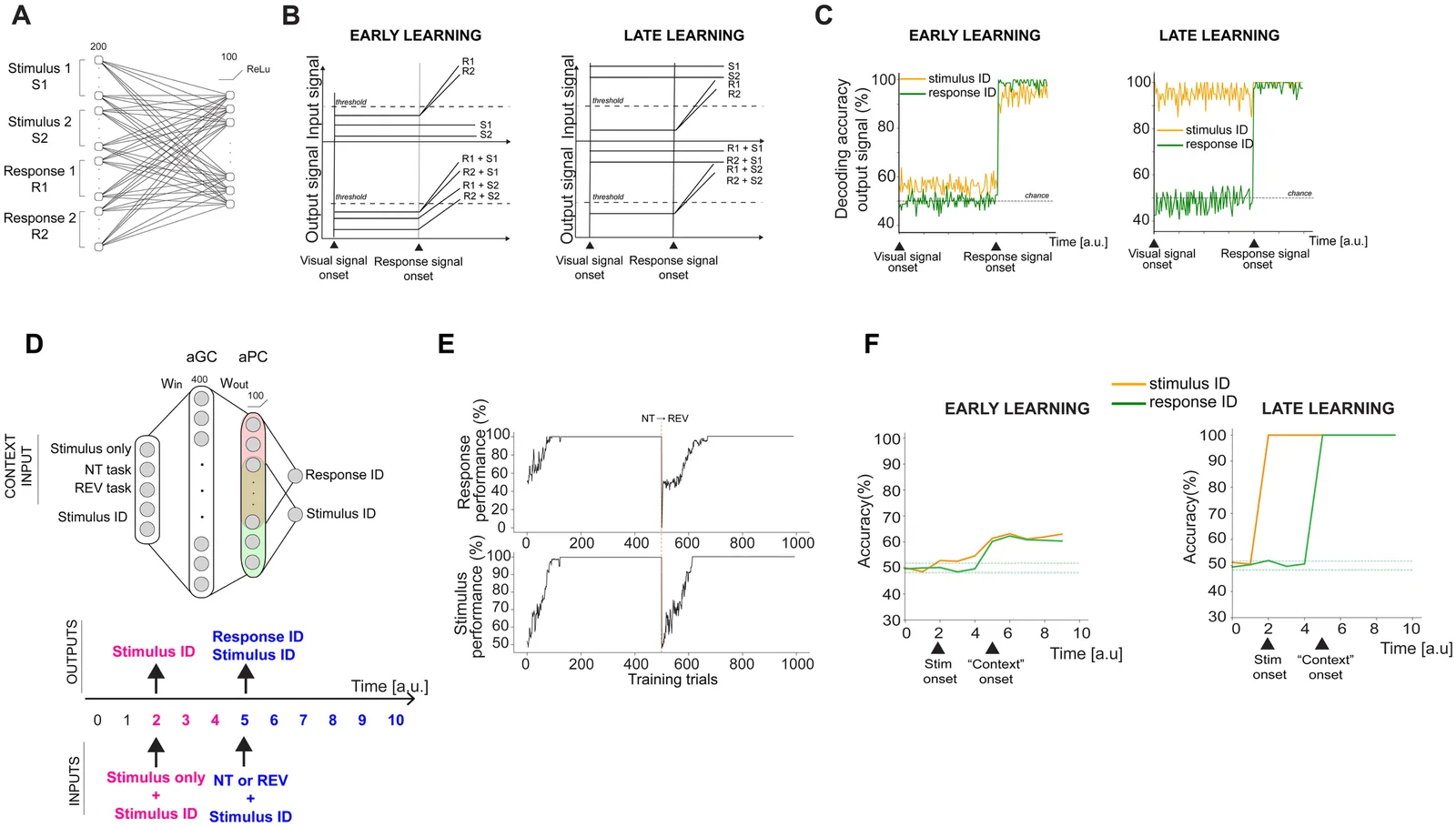
Schema of the input signal to PCs and architecture, performance, and decoding results of the neural network trained to perform the new and reversal tasks. ***A***, Schematic of 200 input units conveying the visual (Stimulus ID) and response (Response ID) inputs. The input units are fully connected to 100 output units. The weights are randomly drawn from a uniform distribution between 0 and 1. The output current from input units is passed through a nonlinearity defined by the rectified linear unit (ReLU). ***B***, Schema of the input (top) and output (bottom) signal during early and late learning. During the early learning trials, the visual signal is below the activation threshold, and it is not decodable from the output activity. ***C***, Decoding accuracy for stimulus and response signal in early and late learning given the input signals modeled as in ***B***. ***D***, Top, Architecture of the feedforward neural network: five input units with a one-hot vector of three units encoding the task context, i.e., stimulus only, NT, or REV, and another one-hot vector of two units encoding the stimulus identity. The input is projected to an expansion layer of 400 units (aGC) through random connection weights (Win) randomly initialized from a uniform distribution with positive values. These input weights are not trained. The output from the aGCs is passed to 100 units layer (aPCs). Two readout units are trained to provide the correct output response and stimulus identity by reading the output activity from two subgroups of aPCs (red and green areas) with an overlap of 20% (yellowish area). The network is trained to provide the correct response associated with the input stimulus and context and the stimulus ID. Bottom, Schematic of the activity of the inputs along a trial. ***E***, Performance of the network during 1,000 training trials. At the 500th trial there is the task switch, from NT to REV task. ***F***, Decoding accuracy of stimulus and response across time from the aPCs activity output during early and late learning. The horizontal dashed lines are 2 standard deviations from the mean of 100 null models. ReLU, rectified linear unit; aGC, artificial granule cell; aPCs, artificial Purkinje cell; NT, novel task; REV, reversal task.

### Model of the input to Purkinje cells

Our aim was to replicate the neural activity of PCs in both the EL and LL after the stimulus onset illustrated in Figure 8*A*,*C*. Indeed, during EL the stimulus signal was decoded after ∼300 ms from the onset of the stimulus (with a brief but significant bump between 200 and 300 ms) almost simultaneously with the response signal (Fig. 8*A*). On the other hand, when the monkeys learned the new association, the neural activity became clearer. The visual signal was decoded after ∼100 ms from the stimulus onset, followed in time by the response signal which was significant after ∼200 ms from stimulus onset (Fig. 8*C*).

We hypothesized that during the early learning stage, the visual input signal to PCs was weak and below the activation threshold of PCs (Fig. 9*A–C*). This made it difficult for the visual input to be decoded by itself without the response input signal, which was above the threshold (Fig. 9*B*, left). However, in the late learning stage, once the visuomotor association was well established, PCs might have received the visual signal above threshold earlier than the response signal, and we could decode the visual signal before the response signal (Fig. 9*B*, right). To test this hypothesis, we developed a model consisting of 200 artificial input units. Out of these units, 50 units each encoded the visual stimulus 1 and 2, and the remaining units encoded response 1 and 2. The input signals were randomly projected to 100 output units with the rectified linear function as the nonlinear activation function (Fig. 9*A*). We decoded the visual and response signals from the activity of the output units, as we did with the neural data. The results showed that during early learning, the visual signal was barely decoded after the stimulus onset, and it became decodable along with the response input (Fig. 9*C*, left). On the other hand, when we provided a stronger visual input above the activation threshold followed in time by the response signal, we observed a different output signal that resembled the neural signal activity observed in the late learning stage of actual data (Fig. 9*C*, right).

### Training of an artificial neural network to perform the new and reversal tasks

We trained a simple feedforward artificial neural network (ANN) to perform the NT and REV tasks. The ANN had five input units, which included a one-hot vector of three units encoding if it were a stimulus-only, the NT, or REV context. The other two units encoded the identity of the stimulus (Fig. 9*D*). The reason for the stimulus-only context input was to reproduce the visual activity after stimulus onset, prior to the response signal. The NT and REV context inputs were necessary because the simple ANN could not achieve high performance on the reversed association, so we were forced to cue the task switch, even though it was not true for the real experiment.

The input was passed through an expansion layer of 400 units (which we refer to as aGCs) for a more biologically plausible interpretation of the model through random connection weights *W*_in_, which were randomly initialized from a uniform distribution with positive values. These input weights were fixed throughout the whole training. The output from the aGCs was passed to a 100 units layer which we labeled as the aPCs layer. The output signal from aGCs was sent to aPCs through *W*_out_ weights that have been trained using a specific learning rule (see Materials and Methods). The network was trained such that an output unit had to provide a binary response (Response ID) associated with the input stimulus and context reading out the activity of 60 aPCs; a second output unit had to provide the visual stimulus identity (Stimulus ID) from the activity of other 60 aPCs, with an overlap of 20% with aPCs units readout from the Response ID unit. This is done in order to disambiguate the stimulus input signal from the output response signal in the aPCs activity (Fig. 9*D*). We trained the ANN to perform the NT and REV tasks in two separate blocks of 500 trials each, for a total of 1,000 training trials. In the first 500 trials, the network was trained to receive the stimulus-only and the NT contexts along with a Stimulus ID randomly chosen in each trial NT task, while in the last 500 trials it was trained to learn the reversed stimulus–response association (REV task). The activity of the input signals were the same as the ones modeled in Figure 9*B*, with the difference that now we did not impose any distinction in the input activity in EL and LL. In particular, we first provided the stimulus-only context and stimulus ID for a few time steps, and then we provided the NT or REV context along with the stimulus ID for the following time bins (Fig. 9*D*, bottom). As before, we modeled a PCs' activity as nonlinear function of the inputs (ReLU). Both stimulus ID and context were randomly chosen at the beginning of each trial. In the case of the stimulus-only input, the network was required to give the stimulus identity as output. In the case of EL and LL, given a random input stimulus and context, the network had to learn the correct response associated to the input stimulus within the input context. After 500 trials, we reversed the stimulus–response association, and we randomly reinitialized 5% of the *W*_out_ weights of aPCs units read out only from the Stimulus ID output unit (Fig. 9*D*), so the network had to learn again to both encode the stimulus-only input and reversed association. Figure 9*E* shows the network's performance in classifying the input stimulus (top row) and the correct response associated with the input stimulus (bottom row) along the training trials. We defined, as done for the real experiment, an early learning stage as the first trials of NT and REV where the performance of the ANN for the correct response was below 60%. We defined the late learning stage as the last 100 trials of NT and REV, where the ANN performance for correctly classifying the response was 100% (Fig. 9*E*).

We decoded the stimulus and the correct response from the output signals of the aPCs using the same analysis tool used on actual data. Figure 9*F* shows the decoding accuracy of the response (green line) and stimulus (orange line): during EL, at time step 2, when the Stimulus ID was provided to the ANN, we could not decode it from the aPCs activity, but only at time step 5 with the response (Fig. 9*F*, left). This temporal separation between the activity evoked by the stimulus and that evoked by the response resembles the neural results we observed in the early learning stage of the monkey, where the stimulus identity was decodable only along with the response signal (Fig. 8*A*). This suggested that, even though the stimulus was provided in the inputs, it was not decodable from the aPCs because it was below the activation threshold (see schematic in Fig. 9*B*, left). Differently, in the late learning phase of the ANN where the performance was 100%, the visual stimulus identity was decodable as soon as it was provided as input to the network, followed in time by the response signal (Fig. 9*F*, right). The modeled activity resembled the neural signal observed in the late learning stage, where the visuomotor association was well established (Fig. 8*C*). This indicated that when the ANN learned the stimulus–response association with high accuracy, the output signal for the stimulus identity was above the aPCs activation threshold and then significantly decodable prior to the onset of the response, as in the actual data.

## Discussion

In the discussion, we address four main points: the timing of choice-related PC activity within the stimulus–response interval; the increasing selectivity of upcoming choice of simple spikes with learning; the shift of complex spikes from reactive to predictive signaling; and the interpretation of these findings within cerebellar forward model theories.

During visuomotor learning, individual PCs modulate at distinct times spanning the stimulus–response interval, collectively encoding the sensorimotor transformation. This population-level organization parallels findings in neocortex, where complex functions are carried by distributed ensembles rather than single neurons (Rigotti et al., 2013; Tye et al., 2024), and echoes cerebellar ensemble coding of saccade timing and direction (Thier et al., 2000; Herzfeld et al., 2015), adaptive pursuit and eyeblink timing (Ten Brinke et al., 2017; Broersen et al., 2023), and task-epoch tiling in dentate neurons (Flierman et al., 2025).

Local circuit models explain how such temporal diversity arises. In the TRACENet framework (De Zeeuw et al., 2023), multimodal mossy fiber inputs converge on granule cells, climbing-fiber error signals induce bidirectional plasticity, and local inhibitory and nucleocortical loops sculpt PC timing (ten Brinke et al., 2015; Gao et al., 2016; Guo et al., 2016; Witter et al., 2016). Consistent with this, we model PCs as nonlinear classifiers integrating granule cell inputs to compute stimulus–response associations (Muscinelli et al.; Dean et al., 2010; Gutierrez-Castellanos et al., 2017; Litwin-Kumar et al., 2017). We propose that early in learning, weak visual inputs require combination with motor signals for discrimination, and with training, visual representations strengthen, shifting encoding earlier and enabling predictive coding in PC ensembles.

Choice-related simple spike activity emerges progressively with learning. In early learning (EL), few PCs discriminate SR-left from SR-right, and population-level discrimination occurs near movement onset—similar for correct and error trials—suggesting a late motor signal rather than a consolidated association. By late learning (LL) and overtraining (OT), more PCs become selective, and population discrimination shifts from perimovement to poststimulus.

This temporal structure is present in other paradigms: precisely timed PC suppressions in eyeblink conditioning (Ten Brinke et al., 2017), predictive simple spike timing in pursuit adaptation (Medina and Lisberger, 2009; Herzfeld et al., 2018), granule cell and nucleocortical feedback advancing PC pauses as a “go” signal (Giovannucci et al., 2017), and Crus I/II PC populations predicting forthcoming actions in sensorimotor association tasks (Deverett et al., 2018; Chabrol et al., 2019; Wagner and Luo, 2020).

Converging evidence confirms a cerebellar role in association learning. Patients with cerebellar lesions show acquisition deficits (Berlijn et al., 2024), Crus II inactivation in macaques abolishes new visuomotor association formation (Sendhilnathan et al., 2024), and optogenetic manipulation of Crus I/II PCs in mice impairs early learning and accuracy in discrimination tasks (Deverett et al., 2019; Bina et al., 2021).

Whether cerebellar cortex remains necessary after consolidation is debated. Patients with cerebellar damage and monkeys with Crus I/II inactivation perform overtrained associations normally (Berlijn et al., 2024; Sendhilnathan et al., 2024), yet optogenetic PC suppression in mice reduces accuracy postlearning (Deverett et al., 2019), LTP/LTD-deficient mice show disrupted acquisition or refinement (Burguière et al., 2010; Bina et al., 2021), and neuroimaging reveals sustained Crus I/II activation during well-established associations (Balsters and Ramnani, 2011). Our finding that Crus I/II PCs encode choice prominently in the overtrained state may appear to contradict our prior inactivation result (Sendhilnathan et al., 2024). We reconcile this by noting that extensive training recruits neurons across both hemispheres and that consolidated memories transfer to the cerebellar nuclei, while savings are retained in the cortex (De Zeeuw and Yeo, 2005; De Zeeuw et al., 2011; Nettekoven et al., 2024). Furthermore, because right-hemisphere PCs respond to both hand choices, each hemisphere alone carries sufficient information to guide the overtrained response and silencing a small cortical region removes only a fraction of a larger bilateral ensemble with nuclear redundancy, leaving execution intact. During early learning, however, only a minority of PCs distinguish left from right with no nuclear transfer yet (De Zeeuw and Yeo, 2005), so inactivating that same region eliminates a critical portion of the nascent choice signal and slows acquisition. Alternatively, the signal observed in the overtrained task may not be a motor signal but instead represents a corollary discharge originating from prefrontal areas, conveying information about the impending choice to facilitate visuomotor association. This is consistent with the finding that unlike the anterior cerebellum, this area does not project to motor cortex, but rather to area prePmd in premotor cortex and the periprincipal cortex (Sendhilnathan et al., 2024).

Complex spikes reorganize with learning. In EL, CSs probability peaks ∼100 ms after stimulus onset. In the same time window, with learning, this peak decreases in both OT and LL. The peak starts to increase ∼150 ms in OT and at ∼180 ms in LL before movement onset. We suggest that given shorter reaction times in OT and in LL, these peaks cannot be related to the movement but reflect a transition from reactive to predictive signaling (Bina et al., 2021). Learning-dependent CS timing shifts have been observed across paradigms: at conditioned response onset in eyeblink conditioning (Ohmae and Medina, 2015; Ten Brinke et al., 2015), suppression of unexpected-reward responses by predictive cues (Heffley and Hull, 2019; Kostadinov et al., 2019), error-linked shifts driving saccadic adaptation (Herzfeld et al., 2018), and conversion of auditory salience into tactile readiness in go/no-go tasks (Bina et al., 2021).

We also observe an inverse CS–SS coupling in OT, where CS peaks coincide with transient SS suppression, consistent with climbing-fiber gating of plasticity to enable timely, goal-directed responses (Bina et al., 2021). In LL we found instead a positive correlation at the peaks where the probability is higher although it is not significant. Thus, CSs evolve from detecting unexpected events to signaling predicted outcomes, shaping SS output as associations consolidate.

In the motor domain, the cerebellum is thought to implement a forward internal model (FIM) that predicts the sensory consequences of motor commands received from motor cortex (Wolpert et al., 1998; Ito, 2000; Shadmehr et al., 2010), enabling fast, precisely timed actions independent of delayed sensory feedback. The FIM is itself acquired through learning: prediction errors between expected and actual sensory outcomes drive error-based plasticity that progressively refines internal predictions (Tanaka et al., 2020), ultimately shifting behavior from feedback-driven to feedforward control. Converging electrophysiological, genetic, imaging, and clinical studies provide strong evidence for this mechanism (Imamizu et al., 2000; Synofzik et al., 2008; Burguière et al., 2010; Popa et al., 2013).

Extending this framework to cognition, it has been proposed that the cerebellum performs analogous predictive computations to support learning and the coordination of neocortical processes (Wagner and Luo, 2020), thereby stabilizing and refining cortical representations during learning. Electrophysiological findings in simple sensorimotor paradigms in mice support this predictive role. Dual-site recordings in mice performing visuomotor tasks revealed that neurons in the dentate nucleus display anticipatory activity predicting preparatory states in premotor cortex well before movement onset (Chabrol et al., 2019). Similarly, activity in the medial cerebellar nucleus precedes cortical signals related to upcoming actions in goal-directed licking tasks (Gao et al., 2018). Optogenetic manipulation of the dentate/interpositus–thalamocortical pathway confirmed the causal role of these predictive signals: activation could trigger, and silencing could block, cue-evoked movements, but only in the learned context, indicating that cerebellar output provides a feedforward timing signal specifying when, but not what, is the movement to initiate (Dacre et al., 2021). Furthermore, granule cells develop predictive responses during eyeblink conditioning (Giovannucci et al., 2017), and cerebellar and premotor ensembles converge onto a shared low-dimensional code during training, reflecting coadaptation of cortical and cerebellar dynamics (Wagner et al., 2019).

Our data extend this framework to arbitrary visuomotor association learning. In EL when the stimulus–response mapping is uncertain, PCs population primarily encodes outcome feedback. Once consolidated, stimulus and movement representations become linked and appear well before the movement, suggesting the emergence of an internal model predicting the correct response. To construct such a model, the cerebellum must receive efference copies of both stimulus and action information. Anatomical studies indicate that this information may be transmitted through reciprocal cortico-cerebello-cortical loops connecting Crus I/II with associative cortical regions, including the dorsolateral prefrontal cortex, supplementary and presupplementary motor areas, the dorsal premotor cortex, and the motor cortex (Middleton and Strick, 2001; Akkal et al., 2007; Hashimoto et al., 2010; Pisano et al., 2021). Within this network, efference copies from the prefrontal cortex likely convey contextual, rule-based, and value-related representations of the visual stimulus (Asaad et al., 1998; Rainer et al., 1998; Wallis and Miller, 2003; McKee et al., 2014; Brincat et al., 2018), whereas those from the premotor cortex transmit motor planning and preparatory information bridging decision and execution (Mitz et al., 1991; Cisek, 2007; Wang et al., 2019).

As experience accumulates, cerebellar circuits construct a FIM linking visual stimuli to specific actions. PCs then integrate efference copies and relay predictive signals to cortical regions via cerebello-thalamo-cortical projections, refining cortical representations and transforming feedback-dependent associations into fast, accurate, context-appropriate behavior.

### Open questions

Several issues remain unresolved. First, PCs may contribute unequally across learning stages, with some acting as “early predictors” and others as “late refiners” (Bina et al., 2021). Second, connectivity differences between Crus I and Crus II PCs (Henschke and Pakan, 2020) may substantially shape their computational repertoires. Third, understanding how heterogeneous PC timing reorganizes dentate nucleus population activity—which integrates both PC output and mossy fiber input—will be critical to complete the circuit picture.
